# Traveling Wave Ion Mobility-Derived Collision Cross Section Database for Plant Specialized Metabolites: An Application to *Ventilago harmandiana* Pierre

**DOI:** 10.1021/acs.jproteome.2c00413

**Published:** 2022-09-26

**Authors:** Narumol Jariyasopit, Suphitcha Limjiasahapong, Alongkorn Kurilung, Sitanan Sartyoungkul, Pattipong Wisanpitayakorn, Narong Nuntasaen, Chutima Kuhakarn, Vichai Reutrakul, Prasat Kittakoop, Yongyut Sirivatanauksorn, Sakda Khoomrung

**Affiliations:** † Metabolomics and Systems Biology, Department of Biochemistry, Faculty of Medicine Siriraj Hospital, Mahidol University, Bangkok 10700, Thailand; ‡ Siriraj Metabolomics and Phenomics Center, Faculty of Medicine Siriraj Hospital, Mahidol University, Bangkok 10700, Thailand; § Center of Excellence for Innovation in Chemistry (PERCH-CIC), Faculty of Science, Mahidol University, Bangkok 10400 Thailand; ∥ Chulabhorn Graduate Institute, Program in Chemical Sciences, Chulabhorn Royal Academy, Laksi, Bangkok 10210, Thailand; ⊥ Chulabhorn Research Institute, Kamphaeng Phet 6 Road, Laksi, Bangkok 10210, Thailand

**Keywords:** *Ventilago harmandiana*, collision cross section, ^TW^CCS_N2_, traveling wave ion mobility, TWIMS, mass spectrometry, metabolomics, natural products

## Abstract

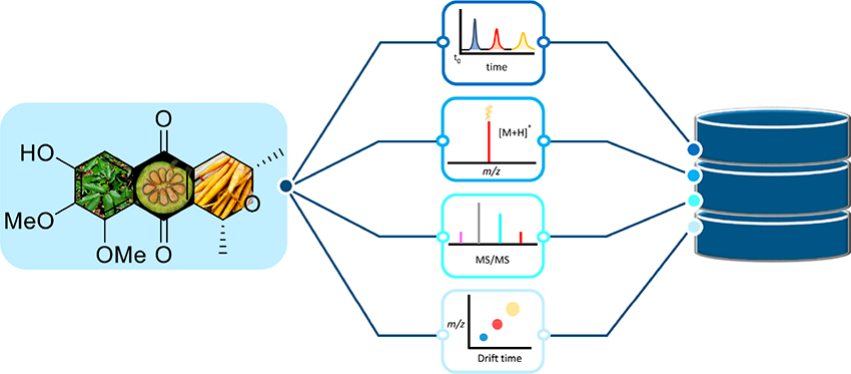

The combination of ion mobility mass spectrometry (IM-MS) and chromatography is a valuable tool for identifying compounds in natural products. In this study, using an ultra-performance liquid chromatography system coupled to a high-resolution quadrupole/traveling wave ion mobility spectrometry/time-of-flight MS (UPLC-TWIMS-QTOF), we have established and validated a comprehensive ^TW^CCS_N2_ and MS database for 112 plant specialized metabolites. The database included 15 compounds that were isolated and purified in-house and are not commercially available. We obtained accurate *m*/*z*, retention times, fragment ions, and TWIMS-derived CCS (^TW^CCS_N2_) values for 207 adducts (ESI^+^ and ESI^–^). The database included novel 158 ^TW^CCS_N2_ values from 79 specialized metabolites. In the presence of plant matrix, the CCS measurement was reproducible and robust. Finally, we demonstrated the application of the database to extend the metabolite coverage of *Ventilago harmandiana* Pierre. In addition to pyranonaphthoquinones, a group of known specialized metabolites in *V. harmandiana*, we identified flavonoids, xanthone, naphthofuran, and protocatechuic acid for the first time through targeted analysis. Interestingly, further investigation using IM-MS of unknown features suggested the presence of organonitrogen compounds and lipid and lipid-like molecules, which is also reported for the first time. Data are available on the MassIVE (https://massive.ucsd.edu, data set identifier MSV000090213).

## Introduction

Plant derived natural products offer a wealth of potential therapeutic candidates. Numerous approved drugs were derived from bioactive metabolites present in plants.^1^ These bioactive metabolites—also referred to as specialized metabolites or secondary metabolites—are produced by a specialized metabolism which is not essential for survival but is a metabolic consequence of environmental adaptation.^2^

Traditional methodology for drug discovery in plants involves isolation and purification of individual compounds from plant extracts that have exhibited biological activities. This methodology encompasses laborious sample preparation and instrumental analyses [e.g., liquid chromatography (LC), mass spectrometry (MS), nuclear magnetic resonance (NMR) spectroscopy]. Over the decades, metabolomics has increasingly been recognized as a valuable technology for research in plants and traditional medicines.^3,4^ Metabolome analysis can provide crucial information about an organism in response to changes due to internal and external perturbations. It has aided in bioactive compound discovery, allowing large-scale metabolite profiling which provides molecular descriptors for the unknowns in complex natural product extracts.

Among these analytical platforms, MS is a mainstream technology for targeted and untargeted analyses because of its high sensitivity and selectivity as well as its ability to provide molecular information. Despite its merits, metabolite identification is challenging in MS-based metabolomics. Various technologies and data analysis strategies have been introduced to overcome such challenges, for example, increasing chromatographic separation dimension,^5,6^ utilizing high-resolution MS or tandem MS,^7,8^ and creating open-access mass spectral databases.^9^ The development of public databases has significantly propelled the application of metabolomics in plant natural products.^10^ Conventionally, the identification of unknown features is achieved by comparing their accurate *m*/*z* values and/or fragment ions (or MS/MS spectra) against mass spectral databases. Despite the advancements in metabolomics technologies, the identification of unknown metabolites in plant extracts still remains a major challenge because they contain a variety of constituents with highly different physicochemical properties and a vast number of isomers.

In recent years, a combination of ion-mobility (IM) and MS (IM-MS) has been increasingly adopted across different research fields of research to improve metabolite identification.^11,12^ IM provides an additional molecular property known as a rotationally averaged collision cross-section (CCS) value that is specific to a molecule and matrix-independent.^13,14^ When combined with chromatography and MS, IM adds another separation dimension, differentiating ions by arrival time, which can improve the quality of mass spectra.^15^ Incorporating CCS values into the traditional MS-based compound identification workflow helps increase metabolite identification confidence. Many studies have created in-house CCS databases to increase the metabolite identification accuracy as well as coverage in biological samples.^13,16,17^ Currently, publicly available CCS databases cover a wide range of compound classes, such as plant metabolites,^18,19^ pesticides,^14,20^ polymers,^19^ and pharmaceuticals.^20^ To facilitate the integration of CCS measurement into the conventional MS-based metabolomics workflows, metabolomics communities have made efforts to create open-access experimental CCS databases, such as the Unified CCS Compendium,^21^ AllCCS,^22^ CCSbase,^23^ and Pacific Northwest National Laboratory,^24^ and to develop CCS prediction models.^22,23,25,26^ Similar to the role of public MS databases, the growing CCS databases will lend support to large-scale metabolite identification. However, a PubMed database search, using keywords “ion mobility mass spectrometry” and “plant”, revealed that in the past 10 years (2012–2022) only 6% of the articles concerning IM-MS are related to plants. In response to the demand for experimental CCS values in plant metabolites, we intend to provide another source of experimental ^TW^CCS_N2_ values, determined by traveling wave ion mobility spectrometry (TWIMS), for specialized metabolites gathered from tropical plants and natural products over the years.

In this study, we aim to (1) develop a method for plant extract analysis using ultra-performance liquid chromatography coupled to a high-resolution quadrupole/TWIMS/time-of-flight MS (UPLC-TWIMS-QTOF), (2) establish a comprehensive MS and TWIMS-derived CCS (^TW^CCS_N2_) database for 112 specialized metabolites, and (3) apply the developed method and database to identify metabolites in *V. harmandiana* and extend the search for other specialized metabolites within this plant. This plant is of particular interest because of its wide spectrum of traditional uses. Medical applications of *Ventilago* include its use in rheumatism, diabetes, and wound infection.^27−29^ Different species of the genus *Ventilago* of the Rhamnaceae family are tropical climbers distributed throughout South Asia and South-East Asia.^30,31^*V. harmandiana* is a rare endemic species found only in Thailand. To date, only ten pyranonaphthoquinones (PNQs) and nine anthraquinones (ATQs) have been identified in *V. harmandiana*, and some of which exhibited anti-inflammatory activities.^28^ Apart from PNQs and ATQs, no other specialized metabolites have been identified. Therefore, we aimed to apply the established ^TW^CCS_N2_ database to explore other metabolites.

## Materials and Methods

### Plant Samples

The details of plant sampling are provided elsewhere.^32^ In brief, leaf, root, bark, wood, and heartwood samples of *V. harmandiana* were collected from Trang Province, Thailand (lat. 7°47′12.8′′ N, long. 99° 30′12.8′′ E; altitude 104 m a.s.l.). The samples were washed with tap water and air-dried, except for the leaves, which were dried in an oven at 80 °C until a constant weight was achieved, before being ground into powder.

### Chemicals

A list of reference standards, sources, and percent (%) purities is given in Table S1. A total of 112 compounds were analyzed: 37 flavonoids, 11 isochromanequinones, 7 linear 1,3-diarylpropanoids, 6 cinnamic acids and derivatives, 18 anthracenes, 12 benzene and substituted derivatives, 10 benzopyrans, 8 prenol lipids, 2 naphthofurans, and 1 organooxygen compound. Among these, 54 metabolites were in-house purified standards with an average % purity of 95%, and 58 metabolites were commercially available standards with a purity ≥98% (Table S1). The commercial reference standards were purchased from Phytolab (Germany), Chemface (China), BioCrick (China), and Sigma-Aldrich (Germany). The in-house purified standards, including isochromanequinones, anthracenes, benzophenones, biphenols, naphthofurans, benzene and substituted derivatives, and flavonoids, were isolated from *V. harmandiana*,^27^*Ventilgo maingayi* M.A. Lawson C (unpublished), *Garcinia speciose* Wall,^33^*Kaempferia parviflora* Wall. ex. Baker *H*,^34^*Kaempferia rotunda* L.,^35^ and *Coccinia grandis* (L.) Voigt. *HC.*(36) We provide simplified molecular-input line-entry system (SMILES) structures of all compounds in Table S1. For the 15 reference standards that were isolated and purified in-house and are not commercially available, their chemical structures and SMILES are provided in Table S2. LC-MS-grade methanol (MeOH), acetonitrile (ACN), and isopropanol (IPA) were purchased from RCI Labscan Limited (Bangkok, Thailand). Formic acid was purchased from Fisher Chemical (U.S.A.). Purified water was obtained using a Milli-Q water system (Milli-Q Advantage A10, Millipore Corporation, France).

### Sample Preparation

All reference standards were prepared in MeOH at 20 μM except for 2,4,6,3′,5′-pentahydroxybenzophenone, 4,6,3′,4′-tetrahydroxy-2-methoxybenzophenone, 2,3′,4,5′-tetrahydroxy-6-methoxybenzophenone, garciosone A, 4,3′,4′-trihydroxy-2,6-dimethoxybenzophenone, garcibiphenyl C, and garciosine A, which were dissolved in water with 0.1% formic acid at the same concentration.

Plant samples were extracted using a previously published method.^32^ Briefly, 30 mg of powdered sample was extracted with 1 mL of MeOH using ultrasonication at 60 °C for 30 min. The crude extract was transferred to a conical tube and the residue was re-extracted with 1 mL of MeOH. The two extracts were combined and dried using a vacuum concentrator (Labconco, MO, U.S.A.). The dried residue was then reconstituted in 1 mL of MeOH. After filtration with hydrophilic poly(vinylidene fluoride), the extract was diluted 10 times prior to LC-IM-MS analysis. Pooled samples (quality control) were prepared by combining 5 μL of the leaf, wood, bark, root, and heartwood extracts and were distributed throughout the sample batches for LC-IM-MS analysis.

### UPLC-TWIMS-QTOF Analysis

All reference standards and plant samples were analyzed on an ACQUITY I-Class UPLC system coupled with a UPLC-TWIMS-QTOFMS (Synapt G2-Si, Waters, U.S.A.) with an electrospray ionization (ESI) interface. Chromatographic separation was achieved on a nonpolar column ACQUITY CSH C18 column (2.1 × 100 mm, 1.7 μM particle size, Waters, Milford, MA). The mobile phase consisted of 0.1% formic acid in water (A) and ACN (B). Using a flow rate of 0.4 mL/min, the gradient elution started with 1% B, increased to 5% within 1 min, increased to 20% within 0.1 min, increased to 40% within 3.9 min, increased to 50% within 5 min, increased to 60% within 1.5 min, increased to 80% within 1.5 min, increased to 90% within 2 min, decreased to 1% within 1 min, and re-equilibrated at 1% B for 3 min.^31^ The column temperature was maintained at 35 °C. The weak and strong needle wash solutions were 5% ACN in H_2_O (v/v) and 1:1:1:1 water/ACN/MeOH/IPA, respectively. The injection volume was 5 μL.

MS conditions were optimized to assist the analysis of the selected specialized metabolites. Capillary voltages were 2.0 kV (negative mode) and 2.5 kV (positive mode). Cone voltages of 20 V (negative mode, ESI^–^) and 30 V (positive mode, ESI^+^) were applied with a cone gas flow rate of 50 L/h. The desolvation gas (N_2_) flow rate was 600 L/h, and the temperature was kept at 200 °C. Source temperature was set at 100 °C. MS was operated in ESI^–^ and ESI^+^ modes to collect mass-to-charge (*m*/*z* ratio) from 30–1000 Da using a scan time of 0.2 s. IM and MS data were acquired in HDMS^E^ mode using MassLynx version 4.1 (Waters Corporation). In the HDMS^E^ mode, MS collects accurate masses of precursor ions and their respective drift times at low collision energies (0 eV), and accurate masses of product ions at high collision energies (energy ramp from 20 to 40 eV). Argon was used as a collision gas. The IM was operated at wave velocities of 800 m/s (ESI^+^) and 1000 m/s (ESI^–^), IM wave height of 30 V, and trap bias of 35 V. Nitrogen was used as a buffer gas in the IM chamber at a flow rate of 60 mL/min. Leucine Enkephalin (part no. 186006013, Waters, U.S.A.) was used to calibrate mass detection by using a reference ion of *m*/*z* 556.2771 (ESI^+^) and 554.2620 (ESI^–^). The TWIMS-derived drift times were calibrated against the Major Mix IMS/TOF Calibration Kit (part no. 186008113, Waters, Wilmslow, UK). All the authentic standards and plant samples were analyzed in triplicates in both ESI^–^ and ESI^+^ modes.

### Intraday and Interday Variations and Spiking Experiments

To evaluate intraday and interday variations in CCS measurements, 10 reference standard solutions were spiked into MeOH, yielding a final concentration of 20 μM. Using the method described above, the standard solutions were analyzed in ESI^+^ and ESI^–^ modes in triplicates within a day for three consecutive days. Furthermore, CCS variations were evaluated in the presence of the matrix by spiking 50 reference standard solutions into the pooled plant matrix at 20 μM. The spiked samples were analyzed in triplicates in ESI^+^ and ESI^–^ modes.

### Data Processing and Analysis

Chemical classification (kingdom, superclass, class, subclass) of the studied compounds was performed using ClassyFire (Table S1).^37^ Progenesis QI Informatics (Nonlinear Dynamics, U.K.) was used to process the acquired HDMS^E^ data. To generate the in-house MS and ^TW^CCS_N2_ database, peak alignment and peak picking were performed before collecting accurate *m*/*z* ratio, retention time, ^TW^CCS_N2_ value, and fragment ions for each reference standard (Tables S1 and S3).

To identify metabolites in plant extracts, raw files were processed in batch in which a QC sample was assigned as a reference for peak alignment. Only those features with an intensity greater than 100 were subjected to metabolite identification. Identified features were classified into four levels based on the Metabolomics Standards Initiative;^38^ level 1 metabolites should be validated with at least two orthogonal data of authentic standards. In this study, level 1 metabolites were identified based on the in-house database using the following criteria: mass error <20 ppm, retention time tolerance <0.1 min, matching experimental fragment ions with in-house fragment ions or those given by in-silico fragmentation embedded in Progenesis QI, and percent CCS difference (ΔCCS%) < 4%. Level 2 metabolites were identified by matching experimental *m*/*z* of adducts with The Human Metabolome Database (HMDB)^39^ and Chemical Entities of Biological Interest (ChEBI) databases,^40^ and matching experimental fragment ions with in-silico fragment ions. Level 4 features carry their *m*/*z* and ^TW^CCS_N2_ values but were unidentified based on the selected databases. Level 3 features are putatively characterized compound classes which were beyond the scope of the current study.

Statistical analysis was performed using Microsoft Excel 2016 (Microsoft). Percent difference in the CCS value (ΔCCS%) compared with the in-house database was calculated using the following equation.
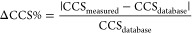


Other percent differences in the CCS values of two compounds of interest (ΔCCS%) were calculated from the equation below.
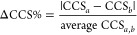


### CCS Prediction

Predicted CCS values were obtained by entering SMILES structures into AllCCS^22^ and CCSbase^23^ web interfaces (Tables S1 and S3). For compounds in which [M – H – H_2_O]^−^ or [M + H – H_2_O]^+^ adducts were observed and their CCS values were not provided by AllCCS and CCSbase, IMoS (Ion Mobility Spectrometry Suite, V1.10c, http://www.imospedia.com) was used to calculate the CCS values.^41^ In brief, three-dimensional structures were generated by Avogadro software^42^ using SMILES. To generate an [M + H – H_2_O]^+^ or [M – H – H_2_O]^−^ adduct, a hydrogen and oxygen atoms were added (protonation) or removed (deprotonation) from the neutral compound using GaussView 5; the charges of [M + H – H_2_O]^+^ and [M – H – H_2_O]^−^ adducts were subsequently verified. The gas-phase geometry optimization calculation of each adduct was performed using Density functional theory (DFT) method at thee B3LYP/6-31+G(d,p) level of theory. All geometrical optimizations were performed using Gaussian 09.^43,44^ The MerzKollman partial charge and dipole moment were calculated using the pop = (mk,dipole) command.^45^ The local minima of each optimized structure were confirmed without imaginary vibrational frequencies. The Gaussian output files (.log) were converted into an IMoS input file (.mfj) using a Python script.^46^ IMoS was used to calculate the average CCS values calculated for each optimized adduct. The CCS calculation was performed using the N2-based trajectory method with Lennard–Jones (TMLJ) parameters and ion-quadrupole potential (QPoL) parameters. According IMoS user manual, the following recommended TMLJ parameters were used: number of orientations = 3, total/orientation = 300 000, time step coefficient = 150, diffuse = 1, temperature = 304 K, and pressure = 101 325 Pa. N_2_ gas and default values for other molecular parameters were selected.^41^

## Results and Discussion

### Natural Product Database Characteristics

**Figure 1:**
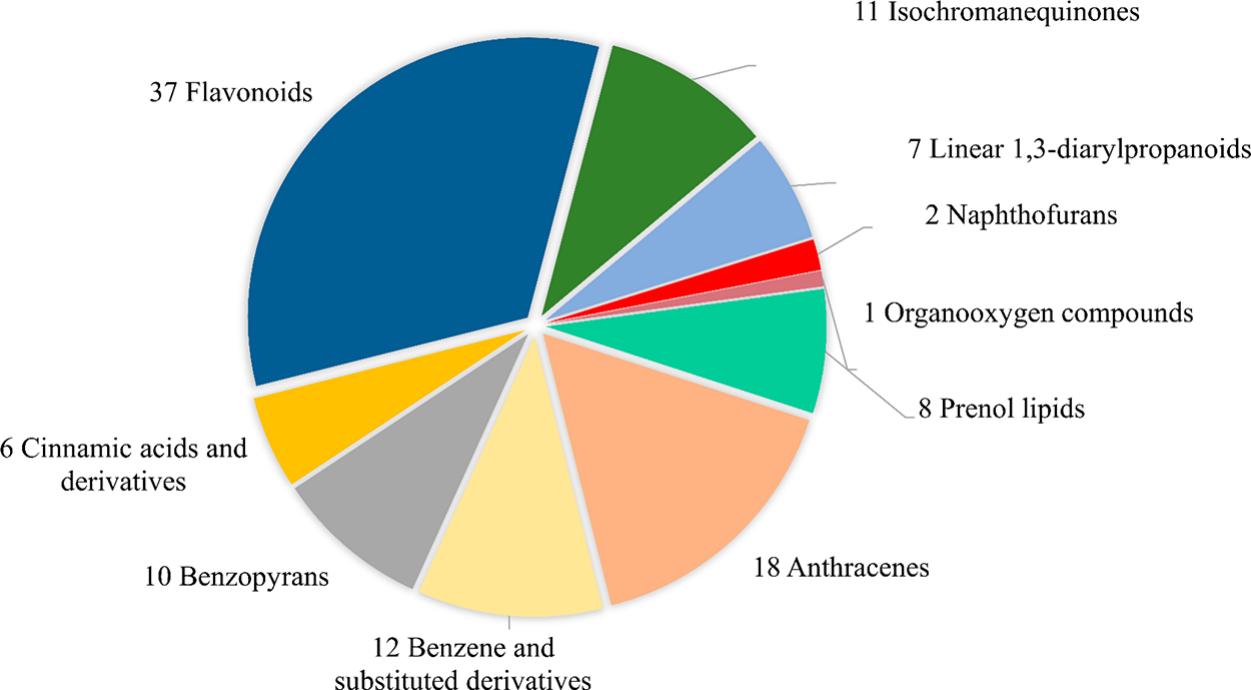
Distribution of compound classes included in the study.

Our in-house MS and ^TW^CCS_N2_ database of specialized metabolites comprised 112 compounds covering masses from 140 to 640 Da, representing 10 classes: 37 flavonoids, 11 isochromanequinones, 7 linear 1,3-diarylpropanoids, 6 cinnamic acids and derivatives, 18 anthracenes, 12 benzene and substituted derivatives, 10 benzopyrans, 8 prenol lipids, 2 naphthofurans, and 1 organooxygen compound (Figure 1). Almost half of the reference standards (48%) were isolated and purified in-house, and 15 of these were not commercially available (Table S2). The current collection contains 21 sets of regioisomers, structural isomers, and stereoisomers.

We obtained the retention times, accurate *m*/*z* values, MS/MS fragment ions, and ^TW^CCS_N2_ values of 207 adducts (ESI^–^ and ESI^+^): 93 [M – H]^−^, 3 [M – H – H_2_O]^−^, 1 [M]^+^, 97 [M + H]^+^, and 13 [M + H – H_2_O]^+^ adducts (Tables S1 and S3). The experimental *m*/*z* values were concentrated in the region between 300 and 350 Da, accounting for 66% of the measured adducts; the ^TW^CCS_N2_ values ranged from 120 to 230 Å^2^. Except for gallic acid, all compounds were detected in the ESI^+^ mode; however, 13 flavonoids, 2 isochromanequinones, and 1 benzene and substituted derivatives were not detected in the ESI^–^ mode. The database included novel 158 ^TW^CCS_N2_ values from 79 specialized metabolites, some of which were PNQs (classified as isochromanequinones) discovered in *V. harmandiana*, triterpenoids (classified as prenol lipids) found in *Ganoderma lucidum*, and naphthofurans found in *V. maingayi*.

To determine the reproducibility of *m*/*z* and CCS measurements, we analyzed all reference standards in triplicate using the ESI- and ESI+ modes. All of the metabolites showed percent relative standard deviations (%RSD) of measured *m/z* values less than 0.001% (Figure S1A). For CCS measurement, 92% of the adducts demonstrated %RSD of less than 2% (Figure S1B). These results indicate that the developed UPLC-TWIMS-QTOF method is robust and reliable. The %RSD < 2% for CCS measurement was relatively larger than those reported by other studies, typically less than 1%.^14,47^ The large deviations could be a result of protomer formation, resulting in multiple conformations of ions in the gas phase. With the low-resolution IM, the presence of unresolved protomers could have fluctuated the CCS values. Protomer formation of polyfunctional compounds was observed in several studies.^48−50^ Recently, caffeine and its isomeric metabolites were investigated using ultrahigh resolution cyclic ion mobility.^51^ As a result, for certain compounds, multiple peaks could be separated using three cycles of IM separation. Our finding calls for cross-laboratory CCS measurements on these plant metabolites—which mostly have never been measured for CCS values—to improve the compound identification using the established database. Taking the experimental CCS data into account, the compound identification was performed using the CCS deviation threshold of <4% in combination with other compound identification criteria including retention time, mass error, and fragment ions.

We further investigated the stability and reproducibility of CCS measurements through intraday and interday experiments on 10 representative metabolites in ESI^–^ and ESI^+^ modes. We chose 5 isomeric pairs to investigate chromatographic, mass, and CCS separations; some of which are present in *V. harmandiana*. The intraday and interday variations were relatively small, with %RSD ranging from <0.01% to 1.8% and 0.2% to 1.4%, respectively (Table S4). The results suggest that the ^TW^CCS_N2_ values were robust and reproducible after the samples were stored for 3 days.

Next, we spiked 50 reference standards from different molecular classes into diluted *V. harmandiana* extract to determine the reducibility of ^TW^CCS_N2_ values in the presence of the plant matrix. For most of the adducts (92%), deviations from the ^TW^CCS_N2_ database (ΔCCS% < 2%) were within the observed uncertainty. The deprotonated adduct of questin yielded the highest ΔCCS% value of 3.5% (Table S5) which could have been due to protomer formation.^51^ Overall, the results demonstrated that the CCS measurement was reproducible and matrix-independent, which is in line with previous studies.^13,14,20^

Among the 50 reference standards, there were three coeluting isomeric pairs: (1) 4,6,3′,4′-tetrahydroxy-2-methoxybenzophenone and 2,3′,4,5′-tetrahydroxy-6-methoxybenzophenone, (2) garciosone A and 4,3′,4′-trihydroxy-2,6-dimethoxybenzophenone, and (3) (+)-gallocatechin and (−)-gallocatechin (Table S6). Overlaid mobiligrams of the three pairs analyzed in ESI^–^ also showed unresolved peaks (Figure S2). The efficiency of resolving two peaks on IM can be measured by percent difference in CCS of two peaks (ΔCCS%), defined as a ratio of difference in CCS of two peaks divided by their average values.^52^ In this context, the ΔCCS% values range from 0.61–1.28 (ESI^+^) and 0.48–0.91 (ESI^–^). Given the small ΔCCS% and chromatographic coelution, an IM with a higher resolving power (R_p_) of at least 130–300 for resolving its isomer peak at half height is required.^52^ Because the mobility separation of these coeluting isomers is limited when using the TWIMS instrument, which has a CCS-based R_p_ of ∼40–50,^52^ they are reported as a sum if present in plant samples. We also calculated the ΔCCS% for the four sets of isomeric pairs spiked in the diluted plant matrix that were accurately identified (Table S6). The ΔCCS% values range from 0 to 0.80 (ESI^+^) and 0.30–2.48 (ESI^–^), which are also extremely small to be baseline separated on TWIMS; however, these isomers were well separated by retention times (Table S6). These results suggest that the combination of chromatography, IM, and MS is indispensable for the analysis of complex samples.

### Correlation of *m*/*z* and ^TW^CCS_N2_ Values

**Figure 2:**
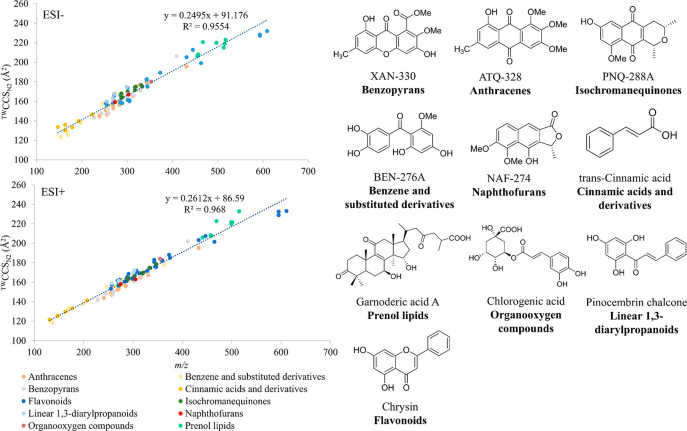
(Left) Correlations of measured *m*/*z* and TWIM-derived CCS values of 112 plant metabolites analyzed in ESI- and ESI+ modes. Dotted lines represent linear fits to the data. (Right) Chemical structures of representative compounds for all classes.

Linear correlations between the measured ^TW^CCS_N2_ and *m*/*z* values were observed for both ESI^–^ and ESI^+^ modes, with correlation coefficients (*R*^2^) of 0.9554 and 0.9680, respectively (Figure 2, left). A representative compound structure for each class is shown in Figure 2 (right). We observed a small deviation from the trendline for the protonated species of prenol lipids (*m*/*z* 440–515 Da) in which the experimental ^TW^CCS_N2_ values were higher than the trendline. Overall, two polarities showed no distinct trendlines among classes, implying that these small metabolites have equally high levels of mass dependency on CCS. Previous studies have observed distinct trendlines for compound classes with higher masses (500–1000 Da), such as lipids and peptides, while small molecules, including specialized metabolites that occupying lower regions of overlap, appear to have overlapping trendlines.^21,24,47,53^ However, Belova et al. observed distinct trendlines of perfluoroalkyl carboxylic and acids and polyfluoroalkyl substances, and other environmental contaminants such as bisphenols, organophosphates, and plasticizers.^17^

To obtain a broader perspective, we overlay the IM-MS associations, categorized into classes, on the Unified CCS Compendium that comprises over 3800 experimental CCS values (Figure S3).^21^ Our collection of experimental CCS values contributed mostly (84%) to the lower region (140–400 Da) of the IM-MS conformational space. Separate overlays of compound classes contained in both databases, which are flavonoids, anthracenes, and benzene and substituted derivatives, show that our ^TW^CCS_N2_ values fit well with the trends deduced from the Unified CCS Compendium (Figure S3, insets).

### CCS Value Comparisons

When considering CCS measurement standardization, the comparability of CCS values derived from different instruments, sample treatment protocols, or laboratories is of concern. An interlaboratory study on CCS values of mycotoxins using TWIMS instruments reported the reproducibility of CCS measurement (ΔCCS% < 2%).^54^ TWIMS-derived CCS values were also compared with drift tube-ion mobility MS (DTIMS)-derived CCS values. Although both TWIMS and DTIMS are considered time-dispersive instruments, DTIMS provides a direct measurement of CCS values while TWIMS provides CCS values that are indirectly obtained based on a calibration procedure where a selection of calibrant can contribute to deviations.^47^ Results from some previous studies supported the comparability of CCS measurements derived by TWIMS and DTIMS, showing small variabilities (∼ΔCCS% < 2%) for compounds such as pesticides, pharmaceuticals, and pesticide metabolites.^20,55^

In this study, we determined the variability of the measured ^TW^CCS_N2_ values and DTIMS-derived CCS (^DT^CCS_N2_) values reported previously (Table S7).^13,16,18,21,24,56−59^ Collectively, 35 database entries, mostly flavonoids, cinnamic acids and derivatives, and benzene and substituted derivatives, comprised ^DT^CCS_N2_ literature values. Of the 35 database entries, 23 and 32 had Δ^DT/TW^CCS% < 2% and <5%, respectively. Deprotonated trans-cinnamic acid yielded the largest Δ^DT/TW^CCS% value of 27%. Owing to the large variation from one source of experimental value, we obtained the predicted values for trans-cinnamic acid using CCSbase and AllCCS. Comparing the predicted values with our value, the results yielded ΔCCS% of 3.8% (CCSbase) and 9.5% (AllCCS), demonstrating that the predicted values are closer to our value than is the experimental ^DT^CCS_N2_ value. However, this large discrepancy requires additional measurements to confirm the absolute value. When the outlier was excluded, the average Δ^DT/TW^CCS% values were 1.3% (standard deviation, SD, 1.4%) and 2.1% (SD 1.7%) for deprotonated and protonated ions, respectively. Comparisons of ^TW^CCS_N2_ values between the measured values and literature CCS values were performed on 26 database entries; 17 and 25 entries had Δ^TW/TW^CCS% < 2% and <5%, respectively (Table S7). The CCS variation was larger for deprotonated ions with an average of 2.8% (SD, 2.3%), but smaller for protonated ions with an average of 1.1% (SD 1.0%). Deviations greater than 5% were observed for deprotonated *p*-coumaric acid (8.0%) and vanillic acid (5.6%). Our ^TW^CCS_N2_ values were systematically larger than those reported previously, which were derived using a different calibrant (polyalanine).^16,57,58^ Variation in TWIM-derived CCS values caused by different calibrants has been previously discussed.^60^ A recent study reported CCS deviation of up to 25% using 11 different calibrants to determine TWIMS-derived CCS values of lipids.^61^ Additionally, different TWIM-MS settings could cause deviations in CCS values,^62,63^ in which, for some compounds, could be larger than a typical ΔCCS% threshold of 2%.

Overall, the percent deviations from ^DT^CCS_N2_ measurements were within the generally accepted CCS deviation (<2%), with the average Δ^DT/TW^CCS% of 1.7 (SD, 1.5) (Table S7). For ^TW^CCS_N2_ measurement, the average Δ^TW/TW^CCS% was 2.2 (SD, 2.0), slightly higher than the typical threshold. Therefore, the results caution the use of ^TW^CCS_N2_ libraries created with different calibrants, and attention must be paid to some outliers when applying this database.

One of the major hurdles in metabolomics is the lack of reference standards to confirm the identity of the features detected in biological samples. Although CCS measurement has been introduced to increase the accuracy of metabolite identification, establishing CCS databases has a similar limitation. Considerable effort has been made on building models to predict CCS values, for instance, CCSbase,^23^ AllCCS,^22^ ISiCLE,^25^ and DeepCCS.^26^ Among them, AllCCS and CCSbase provide a web-based interface and cover diverse molecular classes. In this study, we compared experimental and predicted CCS values obtained by AllCCS and CCSbase. We calculated CCS values using IMoS for compounds for which the CCS values of [M – H – H_2_O]^−^ and [M + H – H_2_O]^+^ adducts were not available in the online databases.

**Figure 3:**
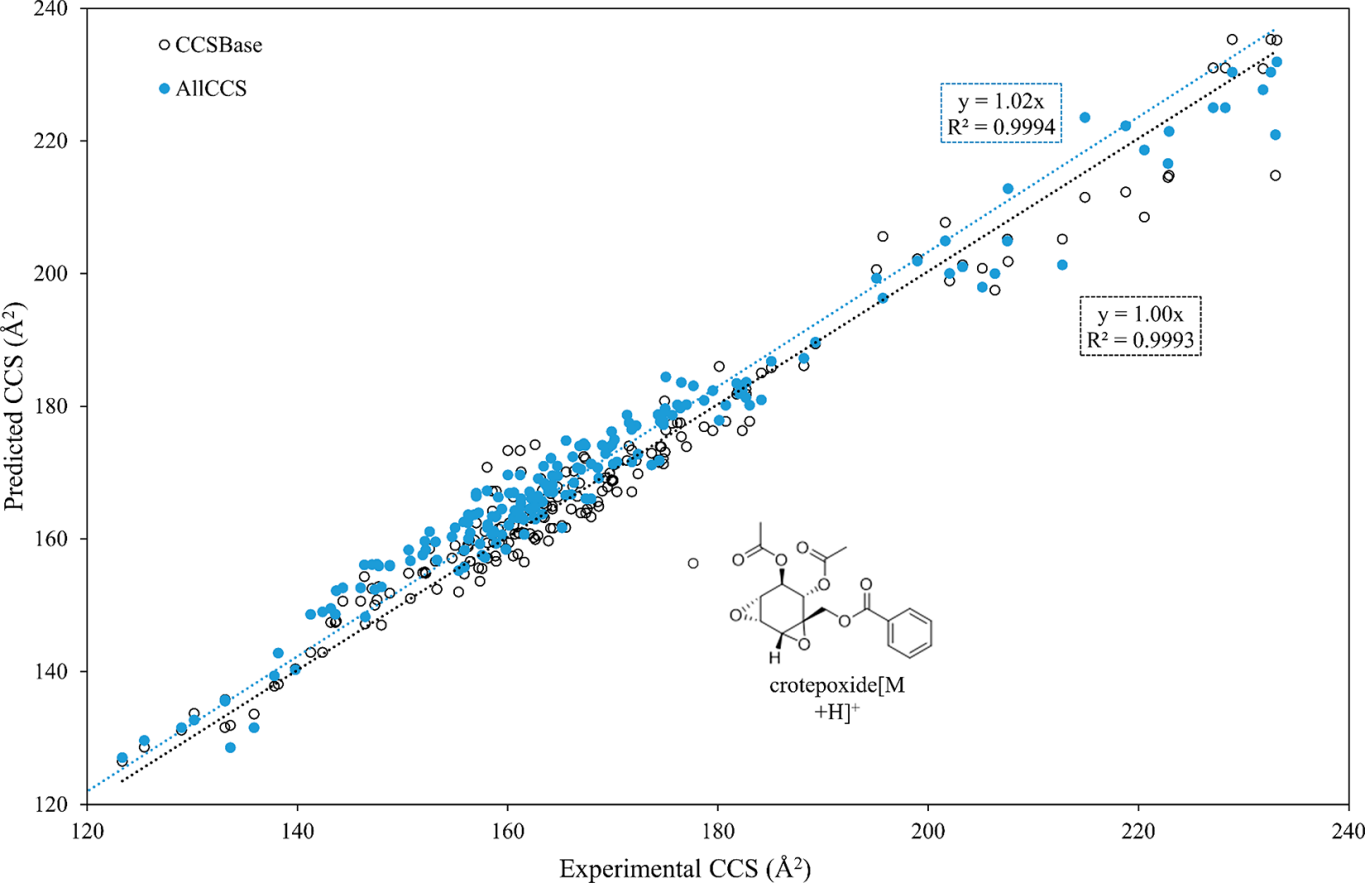
Experimental ^TW^CCS_N2_ values (Å^2^) and predicted values (Å^2^) determined by AllCCS and CCSbase. Dotted lines represent linear fits to the data.

The plots of the experimental and predicted CCS values exhibited good linear relationships (Figure 3). The slopes were 1.02 and 1.00 for AllCCS and CCSbase, respectively, which were close to a perfect fit (slope = 1), implying the highly predictive performance of both models. The linear fits suggest that AllCCS slightly overestimated the CCS values. When comparing the experimental ^TW^CCS_N2_ with the predicted values provided by CCSbase, including all adducts, lower deviations were observed; 64% of the metabolites in the database demonstrated ΔCCS% < 2%, but it was 45% for AllCCS. For isochromanequinones which have never been used to train any models, the predicted values given by CCSbase were closer to the experimental values, with an average ΔCCS% of 1.3% compared with 2.8% given by AllCCS. In this case, the higher predictive performance of CCSbase indicates that it is more generalized; nonetheless, it requires a larger data set with more diverse classes to confirm the observations.

Although CCSbase appears to slightly outperform AllCCS for this set of specialized metabolites, the CCSbase-derived CCS of protonated crotepoxide demonstrated the highest deviation of 12% (Figure 3), indicating that the experimental value was much greater than the predicted value. Crotepoxide, which contains cyclohexane diepoxy functionalities, was the only compound in our database with epoxide groups. This finding is not fully understood. Nonetheless, this outlier suggests that the protonation of this molecular structure may not be well captured by the prediction model, possibly due to its highly oxygenated structure in the presence of reactive epoxides that may form different charged isomers than the predicted protonated structure. The crotepoxide reference standard used in this study was isolated from *Kaempferia rotunda*.^35^

### Extended Metabolite Coverage of *V. harmandiana*

Our previous study quantified six PNQs and two ATQs from leaves, root, bark, wood, and heartwood of *V. harmandiana*.^32^ Because of the therapeutic potentials of *V. harmandiana*,^27^ we were interested in exploring other specialized metabolites using the established database. Therefore, leaf, root, bark, wood, and heartwood samples of *V. harmandiana* were analyzed using the developed UPLC-TWIMS-QTOF along with the developed database. Detected features in the different parts of *V. harmandiana* were categorized into level 1 metabolites, level 2, and level 4 (unknown).

**Figure 4:**
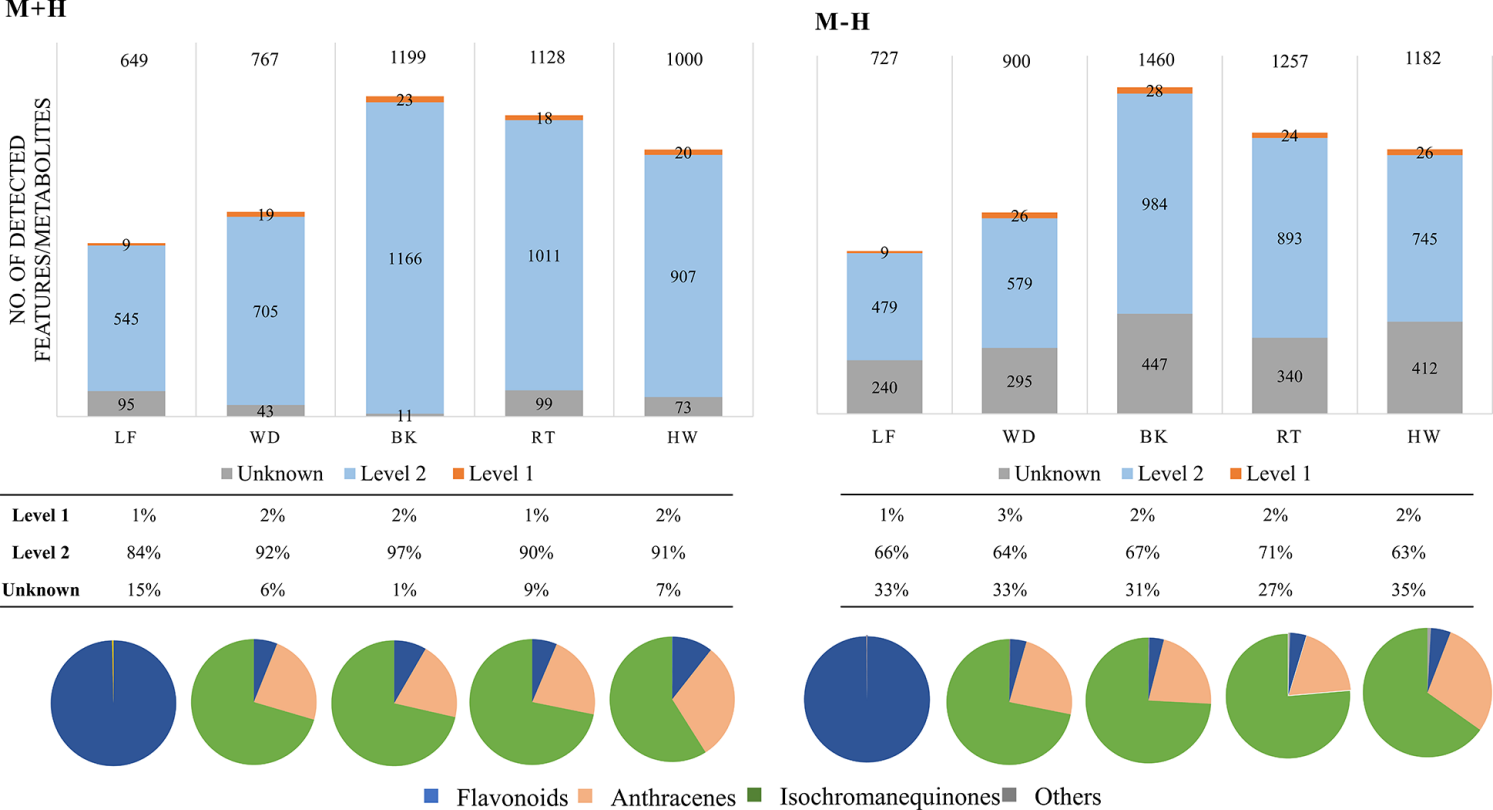
Bar graphs represent the numbers of detected features (protonated and deprotonated adducts) in leaf (LF), wood (WD), bark (BK), root (RT), and heartwood (HW) samples of *V. harmandiana*. The detected features are classified into level 1, level 2, and level 4 (unknown) based on Metabolomics Standards Initiative. Pie charts show distributions of level 1 metabolites, based on their intensities, classified into flavonoids, anthracenes (mostly anthraquinones), isochromanequinones (pyranonaphthoquinones), and others.

The bark samples contained the highest number of features (1199 [M + H]^+^ and 1460 [M – H]^−^ features), while the leaf samples contained the lowest number (649 [M + H]^+^ and 727 [M – H]^−^ features) (Figure 4). In total, 42 level 1 metabolites were identified from all samples, accounting for 1–3% of the total detected features; 29 of these were never detected in *V. harmandiana* (Table S8). Higher intensities were observed in ESI^–^ in which 34 metabolites were identified. In ESI^+^, 32 metabolites were detected, with 8 metabolites that were only identified in this mode: 5,3′-dihydroxy- 3,7,4′-trimethoxyflavone, 5-hydroxy-7,4′-dimethoxyflavone, eriodictyol, naringenin, 5-hydroxy-7-methoxyflavanone, apigenin, pinocembrin chalcone, and PNQ-346. The average mass error range of the identified metabolites was 2.2 ppm (SD 2.6 ppm), and the average ΔCCS% was 0.9% (SD 0.8%).

The distributions of the identified metabolites (level 1) and their intensities in different parts of the plant are shown in Figure 4 (pie charts). The leaf samples exhibited a distinct metabolite profile due to the enrichment of flavonoid glycosides—quercein-3-*O*-rutinoside (rutin) and kaempferol-3-*O*-rutinoside (nicotiflorin). Other parts were enriched in PNQs > ATQs > flavonoids. With regard to PNQs, different distributions were observed across different parts; PNQ-332 was the most abundant PNQ in heartwood and wood, while PNQ-288B and PNQ-302 were most abundant in bark and root, respectively. PNQ-318A, a potent anti-inflammatory metabolite,^27^ was the most abundant in heartwood, followed by bark, wood, and roots (Table S8). To the best of our knowledge, this is the first time that flavonoids XAN-330 and NAF-304 and protocatechuic acid have been identified in *V. harmandiana*.

**Figure 5:**
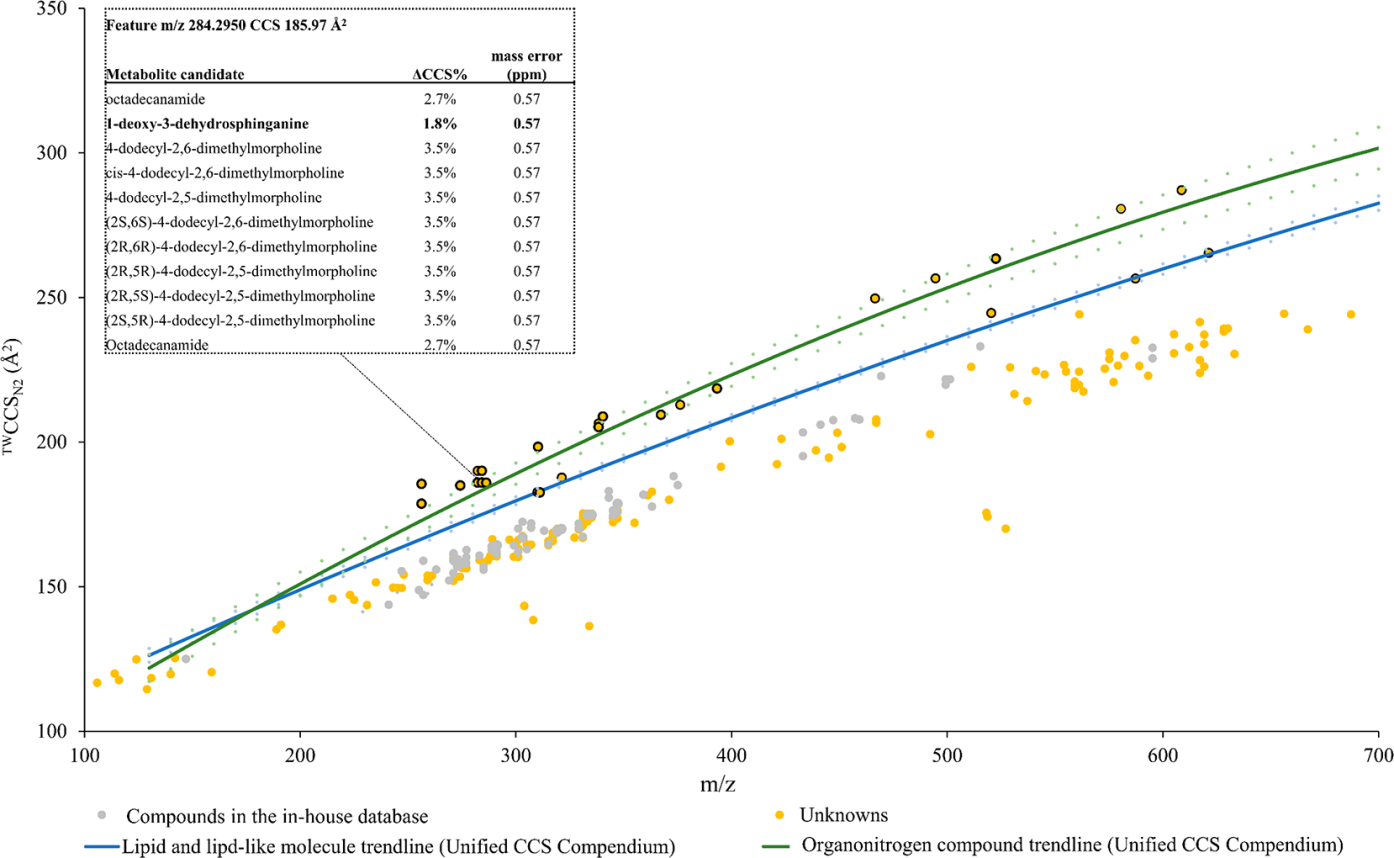
Measured CCS vs *m*/*z* values of protonated adducts of detected features with intensity higher than 1000 in heartwood samples (yellow), compounds in the in-house database (gray). Circled yellow dots represent the features showing higher CCS values than others and were subjected to tentative metabolite identification. Blue and green trendlines (with 95% confidence interval shown in dotted lines) are obtained by regressing CCS and *m*/*z* values of lipid and lipid-like molecules and organonitrogen compounds (Unified CCS Compendium) using a second-order polynomial model.

To further investigate other features through ^TW^CCS_N2_ data, we focused our analysis on heartwood samples because they contained the highest levels of PNQ-318A (Table S8). We plotted the ^TW^CCS_N2_ values and *m*/*z* values of the protonated features that had ion intensities greater than 1000 (Figure 5). The plot shows that most detected features occupied spaces overlapping with the metabolites present in our database where we observed no class-dependent trends. It is also challenging to assign probable class labels using larger databases because the trendlines and corresponding confidence intervals of small molecules overlap. This may require advanced data mining tools to evaluate different trends. In addition, the intrinsic uncertainty associated with CCS measurements can complicate the identification. However, we observed an interesting IM-MS profile of protonated features lying above the others (circled yellow dots in Figure 5), which demonstrated higher ^TW^CCS_N2_ values than other detected features with the same masses. The steeper slope suggests that they belonged to other classes of compounds not present in our database, more likely lipid-like compounds with higher ^TW^CCS_N2_ values due to their less compact structures in the gas phase.^23,47^ By exploring the trendlines provided on the Unified CCS compendium data, another possible class is organonitrogen compounds that also exhibit a steeper trendline. To explore this hypothesis, we overlaid the IM-MS of the detected features with the lipid and lipid-like molecule, and organonitrogen compound trendlines retrieved from the Unified CCS Compendium. Figure 5 shows that the features with higher ^TW^CCS_N2_ values are scattered closer to the organonitrogen compound and lipid and lipid-like molecule trendlines.

To obtain candidate metabolites for those features, we performed metabolite identification by matching the experimental *m*/*z* and fragment ions with the HMDB and ChEBI databases. Using the SMILES of the candidates, we derived the predicted CCS values of potential candidates using CCSbase and AllCCS. For each feature, the candidate with the lowest difference between the average predicted and experimental values (ΔCCS%) was assigned as a tentative metabolite only if ΔCCS% was less than 4% (Table S9). For example, a feature with *m*/*z* 284.2950 and a ^TW^CCS_N2_ value of 185.97 Å^2^ resulted in 11 metabolite candidates. Using SMILES, we obtained the CCS values of the candidates using CCSbase and AllCCS (inset table in Figure 5). This feature was assigned to 1-deoxy-3-dehydrosphinganine which yielded the lowest ΔCCS%. Nonetheless, the accuracy of this metabolite identification approach also depends on the numbers of metabolites listed in the chosen databases. The overall results show that most of the tentative metabolites were fatty acyls, organonitrogen, and organooxygen compounds, which is consistent with the observed ^TW^CCS_N2_ trend. This demonstrated the potential of IM-MS analysis for identifying new metabolites or classes and minimizing the list of metabolite candidates in untargeted experiments.

We repeated a similar search for other parts of the plant samples to report the abundances of these tentative metabolites across the different parts (Table S9). Some of these were isomers in which our experimental data may be insufficient to provide definite structures. As shown in Table S9, five tentative metabolites were consistently detected across the different parts: hexadecanamide and isomers (**1a**,**b**), hexadecasphinganine and an isomer (**3a**,**b**), and 1-deoxysphinganine (**5**) (Table S9). 1-Deoxysphiganine is produced via atypical metabolism by mammals and plants.^64,65^ In humans, the elevated level was linked to many diseases,^66^ and it was found to accumulate when mammalian cells were exposed to a mycotoxin fumonisin B1.^67^ The presence of 1-deoxysphinganine in *V. harmandiana* may lead to future investigations on microbe–plant interactions and associated metabolites.

## Conclusion

We developed an UPLC-TWIMS-QTOF method to characterize 112 plant specialized metabolites including 15 specialized metabolites that were isolated in-house and not commercially available. The established MS and ^TW^CCS_N2_ database provided reliable and reproducible *m*/*z* values, retention times, fragment ions, and ^TW^CCS_N2_ values of 207 adducts (ESI^+^ and ESI^–^). The database includes novel 158 ^TW^CCS_N2_ values from 79 specialized metabolites. We demonstrated that CCS measurement is robust and reliable, yielding small variations when performed within 3 days. The development of ^TW^CCS_N2_ and MS database is ongoing, and we continue to collect data on specialized metabolites from tropical plants.

Regarding the analysis of isomeric compounds, most of the isomeric compounds could be separated by retention times, highlighting the importance of combining chromatography, IM, and MS for the analysis of complex samples. Separating the coeluting isomers with a small ΔCCS% could be restricted by the resolving power of TWIMS. The IM-MS of the specialized metabolites exhibited a good linear relationship; however, we observed no distinct trend among classes. This implied that these specialized metabolites have equally high levels of mass dependency on CCS. Comparability among ^TW^CCS_N2_ and ^DT^CCS_N2_ values are observed, exhibiting small percent deviations (∼ΔCCS% < 2%) for the majority of the selected database entries. For this set of specialized metabolites, CCSbase provided lower prediction errors than AllCCS. However, the highest CCS prediction error for crotepoxide by CCSbase prompted future investigations. To validate the established database, we extended metabolite coverage of *V. harmandiana*. The identified metabolites demonstrated relatively low average mass error (2.2 ± 2.6 ppm) and ΔCCS% (0.9% ± 0.8%). In addition to PNQs, which are important metabolites in *V. harmandiana*, we were able to identify flavonoids, xanthone, naphthofuran, and protocatechuic acid for the first time through targeted analysis. A distinct IM-MS profile of a group of features suggested the presence of organonitrogen compounds and lipid and lipid-like molecules.

## Supporting Information Available

Figures S1–S3; Tables S2, S4–S9 (PDF)
Tables S1 and S3 (XLSX)

## References

[ref1] Newman D. J.; Cragg G. M. Natural products as sources of new drugs over the 30 years from 1981 to 2010. J. Nat. Prod. 2012, 75 (3), 311–335. 10.1021/np200906s. 22316239 PMC3721181

[ref2] Jamwal K.; Bhattacharya S.; Puri S. Plant growth regulator mediated consequences of secondary metabolites in medicinal plants. Journal of applied research on medicinal and aromatic plants 2018, 9, 26–38. 10.1016/j.jarmap.2017.12.003.

[ref3] Hall R. D.; D’Auria J. C.; Silva Ferreira A. C.; Gibon Y.; Kruszka D.; Mishra P.; van de Zedde R. High-throughput plant phenotyping: a role for metabolomics?. Trends Plant Sci. 2022, 27 (6), 549–563. 10.1016/j.tplants.2022.02.001. 35248492

[ref4] Khoomrung S.; Wanichthanarak K.; Nookaew I.; Thamsermsang O.; Seubnooch P.; Laohapand T.; Akarasereenont P. Metabolomics and integrative omics for the development of Thai traditional medicine. Front. Pharmacol. 2017, 8, 474 10.3389/fphar.2017.00474. 28769804 PMC5513896

[ref5] Zhu H.; Wu X.; Huo J.; Hou J.; Long H.; Zhang Z.; Wang B.; Tian M.; Chen K.; Guo D.; Lei M.; Wu W. A five-dimensional data collection strategy for multicomponent discovery and characterization in Traditional Chinese Medicine: Gastrodia Rhizoma as a case study. J. Chromatogr. A 2021, 1653, 462405 10.1016/j.chroma.2021.462405. 34332318

[ref6] Venter P.; Muller M.; Vestner J.; Stander M. A.; Tredoux A. G.; Pasch H.; de Villiers A. Comprehensive three-dimensional LC× LC× ion mobility spectrometry separation combined with high-resolution MS for the analysis of complex samples. Analytical chemistry 2018, 90 (19), 11643–11650. 10.1021/acs.analchem.8b03234. 30193064

[ref7] Perez de Souza L.; Alseekh S.; Scossa F.; Fernie A. R. Ultra-high-performance liquid chromatography high-resolution mass spectrometry variants for metabolomics research. Nat. Methods 2021, 18 (7), 733–746. 10.1038/s41592-021-01116-4. 33972782

[ref8] Rathahao-Paris E.; Alves S.; Junot C.; Tabet J.-C. High resolution mass spectrometry for structural identification of metabolites in metabolomics. Metabolomics 2016, 12 (1), 1–15. 10.1007/s11306-015-0882-8.

[ref9] Misra B. B. New software tools, databases, and resources in metabolomics: Updates from 2020. Metabolomics 2021, 17 (5), 1–24. 10.1007/s11306-021-01796-1. 33977389 PMC8112213

[ref10] Johnson S. R.; Lange B. M. Open-access metabolomics databases for natural product research: present capabilities and future potential. Front. Bioeng. Biotechnol. 2015, 3, 22 10.3389/fbioe.2015.00022. 25789275 PMC4349186

[ref11] Paglia G.; Smith A. J.; Astarita G. Ion mobility mass spectrometry in the omics era: Challenges and opportunities for metabolomics and lipidomics. Mass Spectrom. Rev. 2022, 41 (5), 722–765. 10.1002/mas.21686. 33522625

[ref12] Dodds J. N.; Baker E. S. Ion mobility spectrometry: fundamental concepts, instrumentation, applications, and the road ahead. J. Am. Soc. Mass Spectrom. 2019, 30 (11), 2185–2195. 10.1007/s13361-019-02288-2. 31493234 PMC6832852

[ref13] Stark T. D.; Ranner J.; Stiglbauer B.; Weiss P.; Stark S.; Balemba O. B.; Hofmann T. Construction and application of a database for a five-dimensional identification of natural compounds in *garcinia* species by means of UPLC-ESI-TWIMS-TOF-MS: Introducing gas phase polyphenol conformer drift time distribution intensity ratios. Journal of agricultural and food chemistry 2019, 67 (3), 975–985. 10.1021/acs.jafc.8b06157. 30576604

[ref14] Celma A.; Sancho J. V.; Schymanski E. L.; Fabregat-Safont D.; Ibáñez M.; Goshawk J.; Barknowitz G.; Hernández F. l.; Bijlsma L. Improving target and suspect screening high-resolution mass spectrometry workflows in environmental analysis by ion mobility separation. Environ. Sci. Technol. 2020, 54 (23), 15120–15131. 10.1021/acs.est.0c05713. 33207875

[ref15] Masike K.; Stander M. A.; de Villiers A. Recent applications of ion mobility spectrometry in natural product research. J. Pharm. Biomed. Anal. 2021, 195, 113846 10.1016/j.jpba.2020.113846. 33422832

[ref16] Stander M. A.; Van Wyk B.-E.; Taylor M. J.; Long H. S. Analysis of phenolic compounds in rooibos tea (Aspalathus linearis) with a comparison of flavonoid-based compounds in natural populations of plants from different regions. Journal of agricultural and food chemistry 2017, 65 (47), 10270–10281. 10.1021/acs.jafc.7b03942. 29063755

[ref17] Belova L.; Caballero-Casero N.; van Nuijs A. L.; Covaci A. Ion mobility-high-resolution mass spectrometry (IM-HRMS) for the analysis of contaminants of emerging concern (CECs): Database compilation and application to urine samples. Anal. Chem. 2021, 93 (16), 6428–6436. 10.1021/acs.analchem.1c00142. 33845572

[ref18] Schroeder M.; Meyer S. W.; Heyman H. M.; Barsch A.; Sumner L. W. Generation of a collision cross section library for multi-dimensional plant metabolomics using UHPLC-trapped ion mobility-MS/MS. Metabolites 2020, 10 (1), 13 10.3390/metabo10010013. PMC7023306 31878231

[ref19] Li M.-N.; Wang H.-Y.; Wang R.; Li C.-R.; Shen B.-Q.; Gao W.; Li P.; Yang H. A modified data filtering strategy for targeted characterization of polymers in complex matrixes using drift tube ion mobility-mass spectrometry: Application to analysis of procyanidins in the grape seed extracts. Food Chem. 2020, 321, 126693 10.1016/j.foodchem.2020.126693. 32247183

[ref20] Regueiro J.; Negreira N.; Berntssen M. H. Ion-mobility-derived collision cross section as an additional identification point for multiresidue screening of pesticides in fish feed. Analytical chemistry 2016, 88 (22), 11169–11177. 10.1021/acs.analchem.6b03381. 27779869

[ref21] Picache J. A.; Rose B. S.; Balinski A.; Leaptrot K. L.; Sherrod S. D.; May J. C.; McLean J. A. Collision cross section compendium to annotate and predict multi-omic compound identities. Chemical science 2019, 10 (4), 983–993. 10.1039/C8SC04396E. 30774892 PMC6349024

[ref22] Zhou Z.; Luo M.; Chen X.; Yin Y.; Xiong X.; Wang R.; Zhu Z.-J. Ion mobility collision cross-section atlas for known and unknown metabolite annotation in untargeted metabolomics. Nat. Commun. 2020, 11 (1), 1–13. 10.1038/s41467-020-18171-8. 32859911 PMC7455731

[ref23] Ross D. H.; Cho J. H.; Xu L. Breaking down structural diversity for comprehensive prediction of ion-neutral collision cross sections. Anal. Chem. 2020, 92 (6), 4548–4557. 10.1021/acs.analchem.9b05772. 32096630

[ref24] Zheng X.; Aly N. A.; Zhou Y.; Dupuis K. T.; Bilbao A.; Paurus V. L.; Orton D. J.; Wilson R.; Payne S. H.; Smith R. D.; Baker E. S. A structural examination and collision cross section database for over 500 metabolites and xenobiotics using drift tube ion mobility spectrometry. Chem. Sci. 2017, 8 (11), 7724–7736. 10.1039/C7SC03464D. 29568436 PMC5853271

[ref25] Colby S. M.; Thomas D. G.; Nunez J. R.; Baxter D. J.; Glaesemann K. R.; Brown J. M.; Pirrung M. A.; Govind N.; Teeguarden J. G.; Metz T. O.; Renslow R. S. ISiCLE: a quantum chemistry pipeline for establishing in silico collision cross section libraries. Anal. Chem. 2019, 91 (7), 4346–4356. 10.1021/acs.analchem.8b04567. 30741529 PMC6526953

[ref26] Plante P.-L.; Francovic-Fontaine É.; May J. C.; McLean J. A.; Baker E. S.; Laviolette F.; Marchand M.; Corbeil J. Predicting ion mobility collision cross-sections using a deep neural network: DeepCCS. Analytical chemistry 2019, 91 (8), 5191–5199. 10.1021/acs.analchem.8b05821. 30932474 PMC6628689

[ref27] Panthong K.; Hongthong S.; Kuhakarn C.; Piyachaturawat P.; Suksen K.; Panthong A.; Chiranthanut N.; Kongsaeree P.; Prabpai S.; Nuntasaen N.; Reutrakul V. Pyranonaphthoquinone and anthraquinone derivatives from *Ventilago harmandiana* and their potent anti-inflammatory activity. Phytochemistry 2020, 169, 112182 10.1016/j.phytochem.2019.112182. 31669820

[ref28] Basu S.; Ghosh A.; Hazra B. Evaluation of the antibacterial activity of Ventilago madraspatana Gaertn., Rubia cordifolia Linn. and Lantana camara Linn.: isolation of emodin and physcion as active antibacterial agents. Phytotherapy Research: An International Journal Devoted to Pharmacological and Toxicological Evaluation of Natural Product Derivatives 2005, 19 (10), 888–894. 10.1002/ptr.1752. 16261521

[ref29] Srimoon R.; Anartgnam P.; Tilarux P. In vitro inhibitory efficiency of Ventilago denticulata Willd. dried leaves extract on alpha-glucosidase, alpha-amylase and lipase and antioxidant activities. Sci. Technol. Asia 2020, 135–149.

[ref30] Cooke R.; Johnson B. Colouring matters of Australian plants. XI. Quinones from Ventilago viminalis. Aust. J. Chem. 1963, 16 (4), 695–702. 10.1071/CH9630695.

[ref31] Cahen D.; Utteridge T. M. Three new species of Ventilago (Rhamnaceae) from South-East Asia. Phytotaxa 2017, 307 (3), 171–182. 10.11646/phytotaxa.307.3.1.

[ref32] Limjiasahapong S.; Kaewnarin K.; Jariyasopit N.; Hongthong S.; Nuntasaen N.; Robinson J. L.; Nookaew I.; Sirivatanauksorn Y.; Kuhakarn C.; Reutrakul V.; Khoomrung S. UPLC-ESI-MRM/MS for Absolute Quantification and MS/MS Structural Elucidation of Six Specialized Pyranonaphthoquinone Metabolites From *Ventilago harmandiana*. Front. Plant Sci. 2021, 11, 2038 10.3389/fpls.2020.602993. PMC7830255 33505413

[ref33] Pailee P.; Kuhakarn C.; Sangsuwan C.; Hongthong S.; Piyachaturawat P.; Suksen K.; Jariyawat S.; Akkarawongsapat R.; Limthongkul J.; Napaswad C.; et al. Anti-HIV and cytotoxic biphenyls, benzophenones and xanthones from stems, leaves and twigs of *Garcinia speciosa*. Phytochemistry 2018, 147, 68–79. 10.1016/j.phytochem.2017.12.013. 29304383

[ref34] Jaipetch T.; Reutrakul V.; Tuntiwachwuttikul P.; Santisuk T. Flavonoids in the black rhizomes of Boesenbergia panduta. Phytochemistry 1983, 22 (2), 625–626. 10.1016/0031-9422(83)83075-1.

[ref35] Pancharoen O.; Patrick V.; Reutrakul V.; Tuntiwachwuttikul P.; White A. Constituents of Boesenbergia sp. Isolation and crystal structure of crotepoxide ([1R-(1α, 2α, 4α, 5β, 6α, 7α,)]-4-[(Benzoyloxy) methyl]-3, 8-dioxatricyclo [5, 1, 0, 02, 4] octane-5, 6-diyl diacetate). Australian journal of chemistry 1984, 37 (1), 221–225. 10.1071/CH9840221.

[ref36] Astiti M. A.; Jittmittraphap A.; Leaungwutiwong P.; Chutiwitoonchai N.; Pripdeevech P.; Mahidol C.; Ruchirawat S.; Kittakoop P. LC-QTOF-MS/MS Based Molecular Networking Approach for the Isolation of α-Glucosidase Inhibitors and Virucidal Agents from *Coccinia grandis* (L.) Voigt. Foods 2021, 10 (12), 3041 10.3390/foods10123041. 34945591 PMC8701318

[ref37] Djoumbou Feunang Y.; Eisner R.; Knox C.; Chepelev L.; Hastings J.; Owen G.; Fahy E.; Steinbeck C.; Subramanian S.; Bolton E.; Greiner R.; Wishart D. S. ClassyFire: automated chemical classification with a comprehensive, computable taxonomy. J. Cheminform. 2016, 8 (1), 1–20. 10.1186/s13321-016-0174-y. 27867422 PMC5096306

[ref38] Sumner L. W.; Amberg A.; Barrett D.; Beale M. H.; Beger R.; Daykin C. A.; Fan T. W.-M.; Fiehn O.; Goodacre R.; Griffin J. L.; et al. Proposed minimum reporting standards for chemical analysis. Metabolomics 2007, 3 (3), 211–221. 10.1007/s11306-007-0082-2. 24039616 PMC3772505

[ref39] Wishart D. S.; Guo A.; Oler E.; Wang F.; Anjum A.; Peters H.; Dizon R.; Sayeeda Z.; Tian S.; Lee B. L.; et al. HMDB 5.0: the Human Metabolome Database for 2022. Nucleic Acids Res. 2022, 50 (D1), D622–D631. 10.1093/nar/gkab1062. 34986597 PMC8728138

[ref40] Hastings J.; Owen G.; Dekker A.; Ennis M.; Kale N.; Muthukrishnan V.; Turner S.; Swainston N.; Mendes P.; Steinbeck C. ChEBI in 2016: Improved services and an expanding collection of metabolites. Nucleic acids research 2016, 44 (D1), D1214–D1219. 10.1093/nar/gkv1031. 26467479 PMC4702775

[ref41] Shrivastav V.; Nahin M.; Hogan C. J.; Larriba-Andaluz C. Benchmark comparison for a multi-processing ion mobility calculator in the free molecular regime. J. Am. Soc. Mass Spectrom. 2017, 28 (8), 1540–1551. 10.1007/s13361-017-1661-8. 28477243

[ref42] Hanwell M. D.; Curtis D. E.; Lonie D. C.; Vandermeersch T.; Zurek E.; Hutchison G. R. Avogadro: an advanced semantic chemical editor, visualization, and analysis platform. J. Cheminform. 2012, 4 (1), 1–17. 10.1186/1758-2946-4-17. 22889332 PMC3542060

[ref43] Tirado-Rives J.; Jorgensen W. L. Performance of B3LYP density functional methods for a large set of organic molecules. J. Chem. Theory Comput. 2008, 4 (2), 297–306. 10.1021/ct700248k. 26620661

[ref44] Frisch M. J.; Trucks G. W.; Schlegel H. B.; Scuseria G. E.; Robb M. A.; Cheeseman J. R.; Scalmani G.; Barone V.; Mennucci B.; Petersson G. A.; Nakatsuji H.; Caricato M.; Li X.; Hratchian H. P.; Izmaylov A. F.; Bloino J.; Zheng G.; Sonnenberg J. L.; Hada M.; Ehara M.; Toyota K.; Fukuda R.; Hasegawa J.; Ishida M.; Nakajima T.; Honda Y.; Kitao O.; Nakai H.; Vreven T.; Montgomery J. A. Jr.; Peralta J. E.; Ogliaro F.; Bearpark M.; Heyd J. J.; Brothers E.; Kudin K. N.; Staroverov V. N.; Kobayashi R.; Normand J.; Raghavachari K.; Rendell A.; Burant J. C.; Iyengar S. S.; Tomasi J.; Cossi M.; Rega N.; Millam J. M.; Klene M.; Knox J. E.; Cross J. B.; Bakken V.; Adamo C.; Jaramillo J.; Gomperts R.; Stratmann R. E.; Yazyev O.; Austin A. J.; Cammi R.; Pomelli C.; Ochterski J. W.; Martin R. L.; Morokuma K.; Zakrzewski V. G.; Voth G. A.; Salvador P.; Dannenberg J. J.; Dapprich S.; Daniels A. D.; Farkas Ö.; Foresman J. B.; Ortiz J. V.; Cioslowski J.; Fox D. J. Gaussian 09, Revision A.02; Gaussian, Inc.: Wallingford, CT, 2016.

[ref45] Singh U. C.; Kollman P. A. An approach to computing electrostatic charges for molecules. Journal of computational chemistry 1984, 5 (2), 129–145. 10.1002/jcc.540050204.

[ref46] Ieritano C.; Crouse J.; Campbell J. L.; Hopkins W. S. A parallelized molecular collision cross section package with optimized accuracy and efficiency. Analyst 2019, 144 (5), 1660–1670. 10.1039/C8AN02150C. 30649115

[ref47] Hines K. M.; Ross D. H.; Davidson K. L.; Bush M. F.; Xu L. Large-scale structural characterization of drug and drug-like compounds by high-throughput ion mobility-mass spectrometry. Analytical chemistry 2017, 89 (17), 9023–9030. 10.1021/acs.analchem.7b01709. 28764324 PMC5616088

[ref48] Boschmans J.; Jacobs S.; Williams J. P.; Palmer M.; Richardson K.; Giles K.; Lapthorn C.; Herrebout W. A.; Lemiere F.; Sobott F. Combining density functional theory (DFT) and collision cross-section (CCS) calculations to analyze the gas-phase behaviour of small molecules and their protonation site isomers. Analyst 2016, 141 (13), 4044–4054. 10.1039/C5AN02456K. 27264846

[ref49] McCullagh M.; Douce D.; Van Hoeck E.; Goscinny S. Exploring the complexity of steviol glycosides analysis using ion mobility mass spectrometry. Analytical chemistry 2018, 90 (7), 4585–4595. 10.1021/acs.analchem.7b05002. 29537255

[ref50] Bauer A.; Kuballa J.; Rohn S.; Jantzen E.; Luetjohann J. Evaluation and validation of an ion mobility quadrupole time–of–flight mass spectrometry pesticide screening approach. J. Sep. Sci. 2018, 41 (10), 2178–2187. 10.1002/jssc.201701059. 29446242

[ref51] Sepman H.; Tshepelevitsh S.; Hupatz H.; Kruve A. Protomer Formation Can Aid the Structural Identification of Caffeine Metabolites. Anal. Chem. 2022, 94, 10601 10.1021/acs.analchem.2c00257. 35861491 PMC9352149

[ref52] Dodds J. N.; May J. C.; McLean J. A. Correlating resolving power, resolution, and collision cross section: unifying cross-platform assessment of separation efficiency in ion mobility spectrometry. Analytical chemistry 2017, 89 (22), 12176–12184. 10.1021/acs.analchem.7b02827. 29039942 PMC5744666

[ref53] May J. C.; Goodwin C. R.; Lareau N. M.; Leaptrot K. L.; Morris C. B.; Kurulugama R. T.; Mordehai A.; Klein C.; Barry W.; Darland E.; et al. Conformational ordering of biomolecules in the gas phase: nitrogen collision cross sections measured on a prototype high resolution drift tube ion mobility-mass spectrometer. Anal. Chem. 2014, 86 (4), 2107–2116. 10.1021/ac4038448. 24446877 PMC3931330

[ref54] Righetti L.; Dreolin N.; Celma A.; McCullagh M.; Barknowitz G.; Sancho J. V.; Dall’Asta C. Travelling wave ion mobility-derived collision cross section for mycotoxins: Investigating interlaboratory and interplatform reproducibility. Journal of agricultural and food chemistry 2020, 68 (39), 10937–10943. 10.1021/acs.jafc.0c04498. 32870673 PMC8154562

[ref55] Hinnenkamp V.; Klein J.; Meckelmann S. W.; Balsaa P.; Schmidt T. C.; Schmitz O. J. Comparison of CCS values determined by traveling wave ion mobility mass spectrometry and drift tube ion mobility mass spectrometry. Analytical chemistry 2018, 90 (20), 12042–12050. 10.1021/acs.analchem.8b02711. 30215509

[ref56] Nichols C. M.; Dodds J. N.; Rose B. S.; Picache J. A.; Morris C. B.; Codreanu S. G.; May J. C.; Sherrod S. D.; McLean J. A. Untargeted molecular discovery in primary metabolism: collision cross section as a molecular descriptor in ion mobility-mass spectrometry. Analytical chemistry 2018, 90 (24), 14484–14492. 10.1021/acs.analchem.8b04322. 30449086 PMC6819070

[ref57] Gonzales G. B.; Smagghe G.; Coelus S.; Adriaenssens D.; De Winter K.; Desmet T.; Raes K.; Van Camp J. Collision cross section prediction of deprotonated phenolics in a travelling-wave ion mobility spectrometer using molecular descriptors and chemometrics. Anal. Chim. Acta 2016, 924, 68–76. 10.1016/j.aca.2016.04.020. 27181646

[ref58] Song X.-C.; Canellas E.; Dreolin N.; Nerin C.; Goshawk J. Discovery and Characterization of Phenolic Compounds in Bearberry (Arctostaphylos uva-ursi) Leaves Using Liquid Chromatography–Ion Mobility–High-Resolution Mass Spectrometry. J. Agric. Food Chem. 2021, 69 (37), 10856–10868. 10.1021/acs.jafc.1c02845. 34493038

[ref59] Causon T. J.; Ivanova-Petropulos V.; Petrusheva D.; Bogeva E.; Hann S. Fingerprinting of traditionally produced red wines using liquid chromatography combined with drift tube ion mobility-mass spectrometry. Anal. Chim. Acta 2019, 1052, 179–189. 10.1016/j.aca.2018.11.040. 30685037

[ref60] Gabelica V.; Shvartsburg A. A.; Afonso C.; Barran P.; Benesch J. L.; Bleiholder C.; Bowers M. T.; Bilbao A.; Bush M. F.; Campbell J. L. Recommendations for reporting ion mobility Mass Spectrometry measurements. Mass Spectrom. Rev. 2019, 38 (3), 291–320. 10.1002/mas.21585. 30707468 PMC6618043

[ref61] George A. C.; Schmitz-Afonso I.; Marie V.; Colsch B.; Fenaille F.; Afonso C.; Loutelier-Bourhis C. A re-calibration procedure for interoperable lipid collision cross section values measured by traveling wave ion mobility spectrometry. Anal. Chim. Acta 2022, 1226, 340236 10.1016/j.aca.2022.340236. 36068052

[ref62] Paglia G.; Williams J. P.; Menikarachchi L.; Thompson J. W.; Tyldesley-Worster R.; Halldorsson S.; Rolfsson O.; Moseley A.; Grant D.; Langridge J.; Palsson B. O.; Astarita G. Ion mobility derived collision cross sections to support metabolomics applications. Anal. Chem. 2014, 86 (8), 3985–3993. 10.1021/ac500405x. 24640936 PMC4004193

[ref63] Paglia G.; Angel P.; Williams J. P.; Richardson K.; Olivos H. J.; Thompson J. W.; Menikarachchi L.; Lai S.; Walsh C.; Moseley A.; et al. Ion mobility-derived collision cross section as an additional measure for lipid fingerprinting and identification. Anal. Chem. 2015, 87 (2), 1137–1144. 10.1021/ac503715v. 25495617 PMC4302848

[ref64] Duan J.; Merrill A. H. 1-Deoxysphingolipids encountered exogenously and made de novo: dangerous mysteries inside an enigma. J. Biol. Chem. 2015, 290 (25), 15380–15389. 10.1074/jbc.R115.658823. 25947379 PMC4505451

[ref65] Cahoon R. E.; Solis A. G.; Markham J. E.; Cahoon E. B. Mass Spectrometry-Based Profiling of Plant Sphingolipids from Typical and Aberrant Metabolism. In Plant Lipids; Springer, 2021; Vol. 2295, pp 157–177. 10.1007/978-1-0716-1362-7_10. 34047977

[ref66] Hannich J. T.; Mellal D.; Feng S.; Zumbuehl A.; Riezman H. Structure and conserved function of iso-branched sphingoid bases from the nematode Caenorhabditis elegans. Chemical science 2017, 8 (5), 3676–3686. 10.1039/C6SC04831E. 30155209 PMC6094178

[ref67] Zitomer N. C.; Mitchell T.; Voss K. A.; Bondy G. S.; Pruett S. T.; Garnier-Amblard E. C.; Liebeskind L. S.; Park H.; Wang E.; Sullards M. C.; Merrill A. H.; Riley R. T. Ceramide synthase inhibition by fumonisin B1 causes accumulation of 1-deoxysphinganine: a novel category of bioactive 1-deoxysphingoid bases and 1-deoxydihydroceramides biosynthesized by mammalian cell lines and animals. J. Biol. Chem. 2009, 284 (8), 4786–4795. 10.1074/jbc.M808798200. 19095642 PMC2643501

