# Supplementary material for: Traveling Wave Ion Mobility-Derived Collision Cross Section Database for Plant Specialized Metabolites: An Application to Ventilago harmandiana Pierre
Source: J Proteome Res. 2022 Sep 26;21(10):2481–92. doi: 10.1021/acs.jproteome.2c00413 (PMC9552781; doi:10.1021/acs.jproteome.2c00413)
Supplement: Supplementary file 1 — pr2c00413_si_001.pdf [file pr2c00413_si_001.pdf]

Supporting Information

Travelling Wave Ion Mobility-derived Collision Cross Section Database for Plant Specialized Metabolites: An Application to *Ventilago harmandiana*

Narumol Jariyasopit[1,2], Suphitcha Limjiasahapong[2], Alongkorn Kurilung[1], Sitanan Sartyoungkul[1], Pattipong Wisanpitayakorn[1,2], Chutima Kuhakarn[3], Vichai Reutrakul[3], Prasat Kittakoop[4,5] Yongyut Sirivatanauksorn[2], Sakda Khoomrung[1,2,3,*]

[1]Metabolomics and Systems Biology, Department of Biochemistry, Faculty of Medicine Siriraj Hospital, Mahidol University, Bangkok 10700, Thailand

[2]Siriraj Metabolomics and Phenomics Center, Faculty of Medicine Siriraj Hospital, Mahidol University, Bangkok 10700, Thailand

[3]Center of Excellence for Innovation in Chemistry (PERCH-CIC), Faculty of Science, Mahidol University, Bangkok 10400 Thailand

[4]Chulabhorn Graduate Institute, Program in Chemical Sciences, Chulabhorn Royal Academy, Laksi, Bangkok 10210, Thailand

[5]Chulabhorn Research Institute, Kamphaeng Phet 6 Road, Laksi, Bangkok 10210, Thailand

*Corresponding author: Tel.: +66 2419 5506; fax: +66 2411 0155

E-mail address: sakda.kho@mahidol.edu

Table of Contents

Figure S1. Percent relative standard deviations (%RSD) of measured *m/z* values (Da) and measured $^{TW}CCS_{N2}$CCS values (Å^2)……………………………………………………….......3

Figure S2. Overlaid mobiligrams of three isomeric compounds………………………………4

Figure S3. Measured *m/z* and TWIM-derived CCS values overlaid with those retrieved from Unified CCS Compendium……………………………………………………………………5

Table S2. Chemical and SMILES structures of reference standards isolated in-house and not commercially available……….……………………………………………………………6

Table S4. Percent standard deviation (%RSD) of $^{TW}CCS_{N2}$ values measured in triplicates within a day (intraday) for three consecutive days (interday)………………………………………..9

Table S5. Percent differences between measured $^{TW}CCS_{N2}$ values spiked in diluted plant matrix and those from the database (ΔCCS%)………..…………………………………………………10

Table S6. List of isomeric compounds spiked in diluted plant extracts and their chemical structures……………………………………………………………………………………12

Table S7. Difference between our experimental TWIMS-derived CCS ($^{TW}CCS_{N2}$) values and literature $^{TW}CCS_{N2}$ and $^{DT}CCS_{N2}$ values……………………………………………………...………….15

Table S8. Level 1 metabolites detected in leaf (LF), wood (WD), bark (BK), root (RT), and heartwood (HW) samples of *V. harmandiana* using UPLC-IM-QTOFMS in ESI$^+$ and ESI$^-$ modes……………………18

Table S9. Tentative metabolites identified in heartwood (HW), bark (BK), root (RT), wood (WD), and leaf (LF) samples of *V. harmandiana*………………………………………………………………..21

Figure S1. Percent relative standard deviations (%RSD) of A. measured *m/z* values (Da) and B. measured $^{TW}CCS_{N2}$ values (Å^2) (N = 3) of protonated and deprotonated adducts.

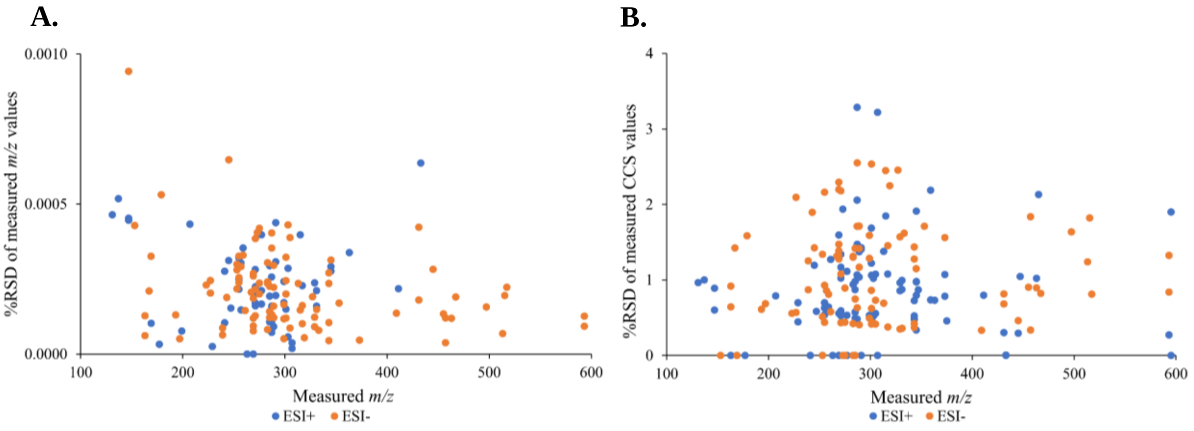

Figure S2. Overlaid mobiligrams of A. 4,6,3',4'-tetrahydroxy-2-methoxybenzophenone/2,3',4,5'-tetrahydroxy-6-methoxybenzophenone, B. garciosone A/4,3',4'-trihydroxy-2,6-dimethoxybenzophenone, and C (+)-gallocatechin/ (-)-gallocatechin standard solutions analyzed in ESI^- mode.

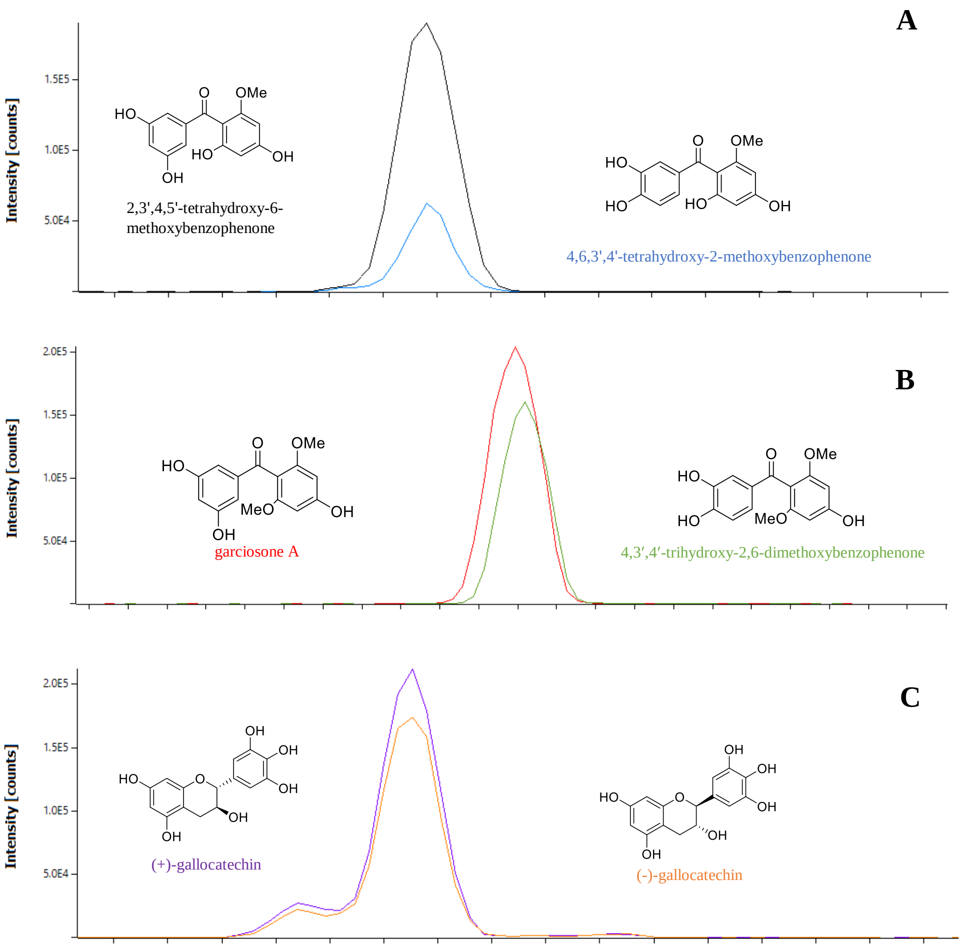

Figure S3. Measured *m/z* and TWIM-derived CCS values determined in this study overlaid with those retrieved from Unified CCS Compendium. Insets are IM-MS plots of chemical classes that are included in both databases.

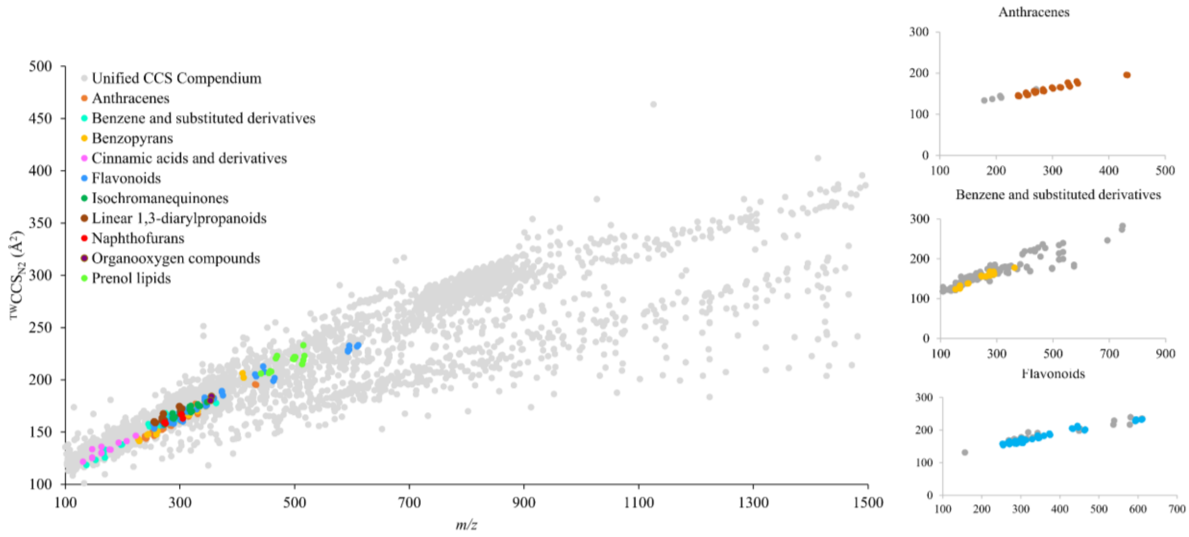

Table S2. Chemical and SMILES structures of reference standards isolated in-house and not commercially available.

No.	Compound	Structure	SMILES
1	XAN-330	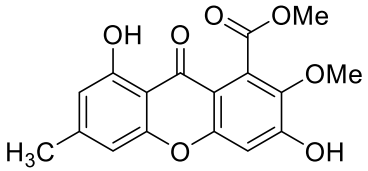	O=C1C2=C(C=C(O)C(OC)=C2C(OC)=O)OC3=CC(C)=CC(O)=C31
2	XAN-344	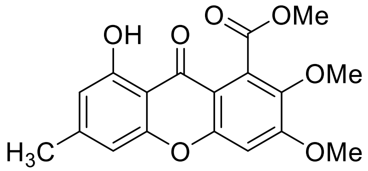	O=C1C2=C(C=C(OC)C(OC)=C2C(OC)=O)OC3=CC(C)=CC(O)=C31
3	ATQ-314	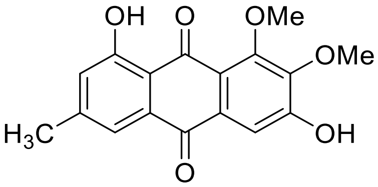	O=C1C2=C(C=C(O)C(OC)=C2OC)C(C3=CC(C)=CC(O)=C31)=O
4	PNQ-288A	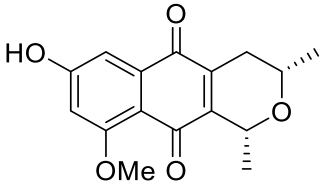	O=C1C2=C([C@@H](C)O[C@@H](C)C2)C(C3=C(OC)C=C(O)C=C31)=O
5	PNQ-288B	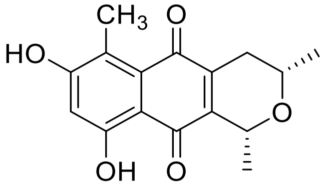	O=C1C2=C([C@@H](C)O[C@@H](C)C2)C(C3=C(O)C=C(O)C(C)=C31)=O

6	PNQ-290	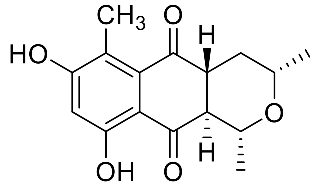	O=C1[C@@]2([H])[C@]([C@@H](C)O[C@@H](C)C2)([H])C(C3=C(O)C=C(O)C(C)=C31)=O
7	PNQ-302	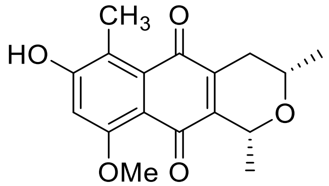	O=C1C2=C([C@@H](C)O[C@@H](C)C2)C(C3=C(OC)C=C(O)C(C)=C31)=O
8	PNQ-318A	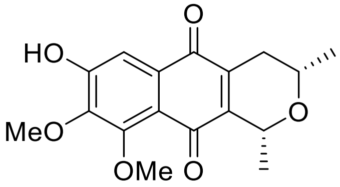	O=C1C2=C([C@@H](C)O[C@@H](C)C2)C(C3=C(OC)C(OC)=C(O)C=C31)=O
9	PNQ-318B	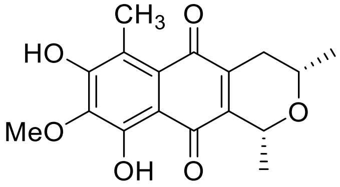	O=C1C2=C([C@@H](C)O[C@@H](C)C2)C(C3=C(O)C(OC)=C(O)C(C)=C31)=O
10	PNQ-320	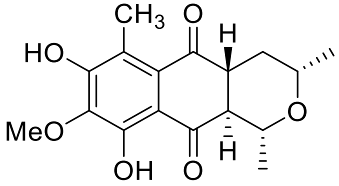	O=C1[C@@]2([H])[C@]([C@@H](C)O[C@@H](C)C2)([H])C(C3=C(O)C(OC)=C(O)C(C)=C31)=O
11	PNQ-332	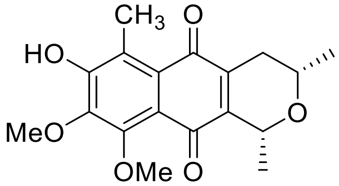	O=C1C2=C([C@@H](C)O[C@@H](C)C2)C(C3=C(OC)C(OC)=C(O)C(C)=C31)=O

12	PNQ-334	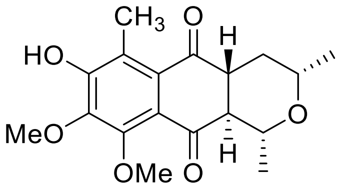	O=C1[C@@]2([H])[C@]([C@@H](C)O[C@@H](C)C2)([H])C(C3=C(OC)C(OC)=C(O)C(C)=C31)=O
13	PNQ-346	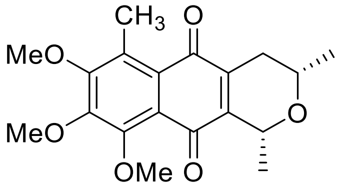	O=C1C2=C([C@@H](C)O[C@@H](C)C2)C(C3=C(OC)C(OC)=C(OC)C(C)=C31)=O
14	NAF-274	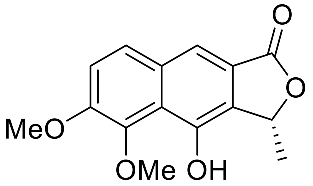	O=C1O[C@H](C)C2=C(O)C3=C(OC)C(OC)=CC=C3C=C21
15	NAF-304	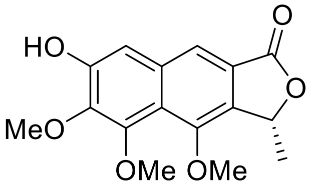	O=C1O[C@H](C)C2=C(OC)C3=C(OC)C(OC)=C(O)C=C3C=C21

Table S4. Percent standard deviation (%RSD) of $^{TW}CCS_{N2}$ values measured in triplicates within a day (intra-day) for three consecutive days (inter-day).

**Compounds**	**Intra-day (n=3)**	**Inter-day (n=3)**
	**%RSD in solvent**	**%RSD in solvent**
**Positive Mode**		
4,6,3',4'-Tetrahydroxy-2-methoxybenzophenone	0.55%	0.36%
2,3',4,5'-Tetrahydroxy-6-methoxybenzophenone	1.08%	0.18%
Garciosone A	0.92%	1.42%
4,3',4'-Trihydroxy-2,6-dimethoxybenzophenone	1.36%	0.17%
1,5-Dihydroxyxanthone	0.67%	0.39%
1,7-Dihydroxyxanthone (Euxanthone)	1.37%	0.96%
Questin	0.55%	0.19%
Physcion	0.55%	0.32%
PNQ-318A	0.48%	0.32%
PNQ-318B	0.48%	0.16%
**Negative Mode**		
4,6,3',4'-Tetrahydroxy-2-methoxybenzophenone	0.42%	0.25%
2,3',4,5'-Tetrahydroxy-6-methoxybenzophenone	0.73%	0.37%
Garciosone A	1.34%	0.22%
4,3',4'-Trihydroxy-2,6-dimethoxybenzophenone	0.00%	0.57%
1,5-Dihydroxyxanthone	1.03%	0.17%
1,7-Dihydroxyxanthone (Euxanthone)	1.83%	0.92%
Questin	0.41%	0.41%
Physcion	< 0.01%	0.24%
PNQ-318A	1.33%	0.21%
PNQ-318B	0.65%	0.87%

Table S5. Percent differences of measured $^{TW}CCS_{N2}$ values (ΔCCS%) spiked in diluted plant matrix and those from the database. %RSD is percent standard deviation of the measured $^{TW}CCS_{N2}$ values (N = 3).

Compound	ESI$^-$			ESI$^+$		
	**Adduct**	**ΔCCS%**	**%RSD**	**Adduct**	**ΔCCS%**	**%RSD**
Chrysophanol	$[M-H]^-$	0.51	0.47	$[M+H]^+$	0.04	0.61
Emodin	$[M-H]^-$	0.03	1.67	$[M+H]^+$	0.12	0.58
Questin	$[M-H]^-$	3.50	0.39	$[M+H]^+$	0.77	0.56
Physcion	$[M-H]^-$	1.47	0.41	$[M+H]^+$	0.13	0.55
Rhein	$[M-H]^-$	1.01	1.15	$[M+H]^+$	0.77	0.00
2-Hydroxyemodin 1-methyl ether	$[M-H]^-$	0.90	1.78	$[M+H]^+$	1.44	0.54
Aurantio-obtusin	$[M-H]^-$	0.49	1.28	$[M+H]^+$	0.34	1.30
2,4,6,3',5'-Pentahydroxybenzophenone	$[M-H]^-$	0.95	0.79	$[M+H]^+$	0.44	0.98
4,6,3',4'-Tetrahydroxy-2-methoxybenzophenone/2,3',4,5'-Tetrahydroxy-6-methoxybenzophenone*	$[M-H]^-$	0.09	0.43	$[M+H]^+$	0.78	0.95
Garciosone A/4,3',4'-Trihydroxy-2,6-dimethoxybenzophenone*	$[M-H]^-$	0.81	0.39	$[M+H]^+$	0.42	1.04
Chrysin	$[M-H]^-$	2.06	3.15	$[M+H]^+$	0.42	1.00
Pinocembrin	$[M-H]^-$	1.21	2.01	$[M+H]^+$	1.59	2.05
Apigenin	$[M-H]^-$	0.24	1.57	$[M+H]^+$	1.32	0.55
Luteolin	$[M-H]^-$	1.25	1.12	$[M+H]^+$	0.02	0.53
Kaempferol	$[M-H]^-$	1.99	0.72	$[M+H]^+$	0.64	0.53
(+)-Gallocatechin/(-)-Gallocatechin*	$[M-H]^-$	0.13	0.42	$[M+H]^+$	1.06	2.05
trans-Cinnamic acid	$[M-H]^-$	0.67	0.61	$[M+H-H_2O]^+$	0.03	0.00
p-Coumaric acid	$[M-H]^-$	1.80	0.62	$[M+H-H_2O]^+$	0.05	0.89
Vanillic acid	$[M-H]^-$	0.73	0.00	$[M+H]^+$	0.52	0.00
Caffeic acid	$[M-H]^-$	0.30	0.00	$[M+H-H_2O]^+$	0.54	0.00
Sinapic acid	$[M-H]^-$	1.02	1.30	$[M+H-H_2O]^+$	0.49	0.70
Chlorogenic acid	$[M-H]^-$	0.92	0.57	$[M+H]^+$	0.27	0.00
PNQ-288A	$[M-H]^-$	0.56	1.42	$[M+H]^+$	0.54	0.52
PNQ-290	$[M-H]^-$	1.73	0.39	$[M+H]^+$	0.84	0.52
PNQ-318A	$[M-H]^-$	0.75	0.37	$[M+H]^+$	0.56	0.49
PNQ-318B	$[M-H]^-$	0.34	1.36	$[M+H]^+$	1.12	0.49
PNQ-320	$[M-H]^-$	0.53	1.31	$[M+H]^+$	0.56	0.48
PNQ-332	$[M-H]^-$	1.11	0.92	$[M+H]^+$	1.35	1.40
PNQ-334	$[M-H]^-$	2.75	1.18	$[M+H]^+$	0.55	0.80
Lucidenic acid A	$[M-H]^-$	2.84	0.25	$[M+H-H_2O]^+$	1.09	0.99
Lucidenic acid B	$[M-H-H_2O]^-$	1.39	1.85	$[M+H-H_2O]^+$	0.11	0.33
Ganoderic acid B	$[M-H-H_2O]^-$	0.40	0.40	$[M+H-H_2O]^+$	0.73	0.51
Ganoderic acid C2	$[M-H]^-$	1.46	0.40	$[M+H-H_2O]^+$	0.37	0.50

Compound	ESI$^-$			ESI$^+$		
	**Adduct**	**ΔCCS%**	**%RSD**	**Adduct**	**ΔCCS%**	**%RSD**
Ganoderic acid DM	$[M-H]^-$	0.68	2.05	$[M+H]^+$	1.16	2.09
Ganoderenic acid A	$[M-H]^-$	0.98	1.24	$[M+H]^+$	0.45	0.53
1,5-Dihydroxyxanthone	$[M-H]^-$	0.39	0.90	$[M+H]^+$	0.16	0.52
1,7-Dihydroxyxanthone (Euxanthone)	$[M-H]^-$	1.89	0.51	$[M+H]^+$	0.14	1.36
1,3,5-Trihydroxyxanthone	$[M-H]^-$	1.66	1.54	$[M+H]^+$	0.45	0.79
1,6-Dihydroxy-5-methoxyxanthone (Buchanaxanthone)	$[M-H]^-$	0.62	1.23	$[M+H]^+$	0.57	2.70
4-Hydroxyvertixanthone	$[M-H]^-$	2.41	1.39	$[M+H]^+$	0.55	0.69
Conioxanthone A	$[M-H]^-$	3.01	1.63	$[M+H]^+$	0.16	0.67
XAN-330	$[M-H]^-$	0.77	2.61	$[M+H]^+$	1.02	0.68
Catechin	$[M-H]^-$	0.06	0.43	$[M+H]^+$	0.73	0.63
Quercetin	$[M-H]^-$	0.20	0.00	$[M+H]^+$	2.12	1.03
Eupatorin	$[M-H]^-$	0.19	0.87	$[M+H]^+$	0.09	0.53
Syringetin	$[M-H]^-$	0.23	0.62	$[M+H]^+$	3.14	0.00
Isoquercitrin	$[M-H]^-$	0.22	0.73	$[M+H]^+$	1.12	1.30

* Co-eluted isomeric compounds are reported as sums.

Table S6. List of isomeric compounds spiked in diluted plant extracts and their chemical structures.

Group	Compound	Chemical structure	Retention time (min)	$[M\text{-}H]^-$		$[M+H]^+$	
				**Measured *m/z***	$^{TW}CCS_{N2}$ **(Å²)**	**Measured *m/z***	$^{TW}CCS_{N2}$ **(Å²)**
A	Questin	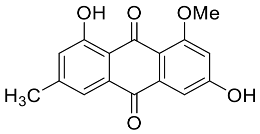	6.55	283.0627	157.02	285.0781	156.86
A	Physcion	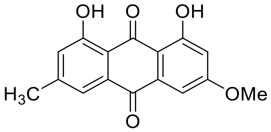	12.10	283.0600	160.16	285.0760	156.86
A	Rhein	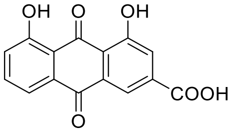	7.80	283.0256	156.23	285.0403	155.84
B	Luteolin	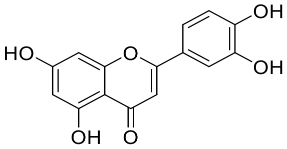	4.09	285.0423	158.54	287.0572	161.11
B	Kaempferol	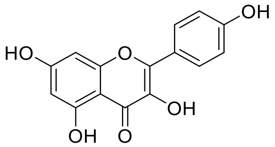	4.98	285.0435	158.92	287.0573	162.12
C	PNQ-318A	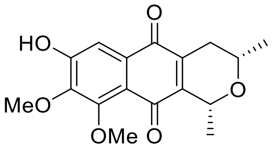	5.89	317.1058	174.31	319.1191	169.02

Group	Compound	Chemical structure	Retention time (min)	$[M-H]^-$		$[M+H]^+$	
				**Measured *m/z***	**$^{TW}CCS_{N2}$ (Å^2)**	**Measured *m/z***	**$^{TW}CCS_{N2}$ (Å^2)**
C	PNQ-318B	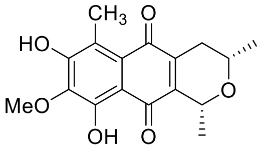	10.30	317.1057	171.37	319.1182	169.50
D	1,5-Dihydroxyxanthone	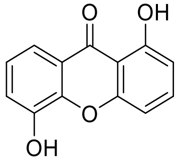	6.12	227.0375	143.61	229.0504	141.27
D	1,7-Dihydroxyxanthone (Euxanthone)	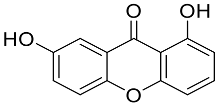	6.50	227.0371	143.18	229.0515	142.40
E	4,6,3',4'-Tetrahydroxy-2-ethoxybenzophenone	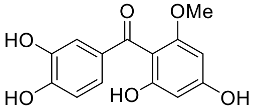	2.81	275.0565	158.68	277.0710	160.12
E	2,3',4,5'-Tetrahydroxy-6-methoxybenzophenone	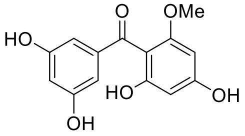	2.81	275.0581	159.45	277.0724	158.09
F	Garciosone A	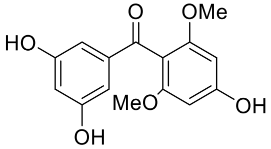	2.98	289.0745	167.57	291.0874	161.27
F	4,3',4'-Trihydroxy-2,6-imethoxybenzophenone	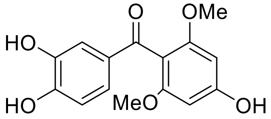	2.98	289.0743	166.05	291.0873	162.26

Group	Compound	Chemical structure	Retention time (min)	[M-H]$^-$		[M+H]$^+$	
				**Measured *m/z***	**$^{TW}CCS_{N2}$ (Å^2)**	**Measured *m/z***	**$^{TW}CCS_{N2}$ (Å^2)**
G	(+)-Gallocatechin	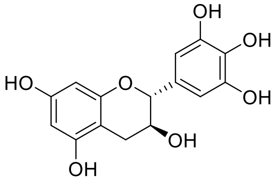	2.03	305.0677	161.19	307.0823	171.82
G	(-)-Gallocatechin	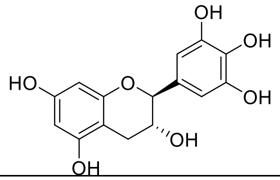	2.03	305.0671	160.03	307.0820	170.40

Table S7. Difference between our experimental TWIMS-derived CCS ($^{TW}CCS_{N2}$) values and literature $^{TW}CCS_{N2}$ and $^{DT}CCS_{N2}$ values. $\Delta^{DT/TW}CCS\%$ is calculated by the difference between $^{TW}CCS_{N2}$ and $^{DT}CCS_{N2}$ divided by average of $^{TW}CCS_{N2}$ and $^{DT}CCS_{N2}$. $\Delta^{TW/TW}CCS\%$ is calculated by the difference between $^{TW}CCS_{N2}$ and $^{TW}CCS_{N2}$ divided by average of $^{TW}CCS_{N2}$.

**Compound**	**Adduct**	**$\Delta^{DT/TW}CCS\%$**	**$\Delta^{TW/TW}CCS\%$**
Emodin	$[M-H]^-$	2.67	
trans-Cinnamic acid	$[M-H]^-$	26.69	
Protocatechuic acid	$[M-H]^-$	0.61	3.92
p-Coumaric acid	$[M-H]^-$	0.55	7.95
Vanillic acid	$[M-H]^-$	5.64	5.64
Gallic acid	$[M-H]^-$	0.01	4.94
Caffeic acid	$[M-H]^-$	0.28	
Ferulic acid	$[M-H]^-$	0.18	3.75
Syringic acid	$[M-H]^-$		0.38
Sinapic acid	$[M-H]^-$		0.52
Chlorogenic acid	$[M-H]^-$	3.40	0.69
Chrysin	$[M-H]^-$	1.35	
Apigenin	$[M-H]^-$	0.95	4.89
Naringenin	$[M-H]^-$	0.10	
Sakuranetin	$[M-H]^-$	1.05	
Luteolin	$[M-H]^-$	1.07	2.20
Kaempferol	$[M-H]^-$	2.59	1.84
Eriodictyol	$[M-H]^-$	1.42	
Catechin	$[M-H]^-$	0.14	1.61
Quercetin	$[M-H]^-$	1.44	1.80
Hesperetin	$[M-H]^-$	1.44	3.36
Isoquercitrin	$[M-H]^-$		0.56
Quercetin-3-O-rutinoside	$[M-H]^-$	0.45	0.11
α-Mangostin	$[M+H]^+$	1.61	1.61
1,8-Dihydroxyanthraquinone (Danthron)	$[M+H]^+$		3.14
Emodin	$[M+H]^+$	6.15	
Protocatechuic acid	$[M+H-H_2O]^+$	3.20	
Vanillic acid	$[M+H]^+$	2.32	
Caffeic acid	$[M+H-H_2O]^+$	0.12	
Chlorogenic acid	$[M+H]^+$	0.74	
Chrysin	$[M+H]^+$	1.04	1.04
5-Hydroxy-7-methoxyflavanone	$[M+H]^+$	2.05	
Apigenin	$[M+H]^+$		0.29
5,7-Dimethoxyflavone	$[M+H]^+$		0.14
Kaempferol	$[M+H]^+$	1.28	1.82
Cyanidin	$[M]^+$	3.07	
Eriodictyol	$[M+H]^+$	2.73	
Catechin	$[M+H]^+$	0.11	

Compound	Adduct	$\Delta^{DT/TW}$CCS%	$\Delta^{TW/TW}$CCS%
Quercetin	$[M+H]^+$	1.88	0.19
Hesperetin	$[M+H]^+$	4.34	0.31
5-Hydroxy-3,7,3',4'-tetramethoxyflavone	$[M+H]^+$		1.05
Quercetin-3-O-rutinoside	$[M+H]^+$	0.61	1.07
Average (SD) excluding outlier (trans-Cinnamic acid)		1.66 (1.54)	2.17 (2.00)

Literature values of $^{TW}CCS_{N2}$ were taken from the following studies,

Gonzales, G. B.; Smagghe, G.; Coelus, S.; Adriaenssens, D.; De Winter, K.; Desmet, T.; Raes, K.; Van Camp, J., Collision cross section prediction of deprotonated phenolics in a travelling-wave ion mobility spectrometer using molecular descriptors and chemometrics. *Analytica Chimica Acta* **2016,** *924*, 68-76.

Stander, M. A.; Van Wyk, B.-E.; Taylor, M. J.; Long, H. S., Analysis of phenolic compounds in rooibos tea (Aspalathus linearis) with a comparison of flavonoid-based compounds in natural populations of plants from different regions. Journal of agricultural and food chemistry 2017, 65 (47), 10270-10281.

Stark, T. D.; Ranner, J.; Stiglbauer, B.; Weiss, P.; Stark, S.; Balemba, O. B.; Hofmann, T., Construction and application of a database for a five-dimensional identification of natural compounds in garcinia species by means of UPLC-ESI-TWIMS-TOF-MS: Introducing gas phase polyphenol conformer drift time distribution intensity ratios. Journal of agricultural and food chemistry 2018, 67 (3), 975-985.

Song, X.-C.; Canellas, E.; Dreolin, N.; Nerin, C.; Goshawk, J., Discovery and Characterization of Phenolic Compounds in Bearberry (Arctostaphylos uva-ursi) Leaves Using Liquid Chromatography–Ion Mobility–High-Resolution Mass Spectrometry. Journal of Agricultural and Food Chemistry 2021, 69 (37), 10856-10868.

Hines, K. M.; Ross, D. H.; Davidson, K. L.; Bush, M. F.; Xu, L., Large-scale structural characterization of drug and drug-like compounds by high-throughput ion mobility-mass spectrometry. Analytical chemistry 2017, 89 (17), 9023-9030.

Literature values of $^{DT}CCS_{N2}$ were taken from the following studies,

Zheng, X.; Aly, N. A.; Zhou, Y.; Dupuis, K. T.; Bilbao, A.; Paurus, V. L.; Orton, D. J.; Wilson, R.; Payne, S. H.; Smith, R. D., A structural examination and collision cross section database for over 500 metabolites and xenobiotics using drift tube ion mobility spectrometry. Chemical Science 2017, 8 (11), 7724-7736.

Causon, T. J.; Ivanova-Petropulos, V.; Petrusheva, D.; Bogeva, E.; Hann, S., Fingerprinting of traditionally produced red wines using liquid chromatography combined with drift tube ion mobility-mass spectrometry. Analytica Chimica Acta 2019, 1052, 179-189.

Picache, J. A.; Rose, B. S.; Balinski, A.; Leaptrot, K. L.; Sherrod, S. D.; May, J. C.; McLean, J. A., Collision cross section compendium to annotate and predict multi-omic compound identities. Chemical science 2019, 10 (4), 983-993.

Zhou, Z.; Luo, M.; Chen, X.; Yin, Y.; Xiong, X.; Wang, R.; Zhu, Z.-J., Ion mobility collision cross-section atlas for known and unknown metabolite annotation in untargeted metabolomics. Nature communications 2020, 11 (1), 1-13.

Nichols, C. M.; Dodds, J. N.; Rose, B. S.; Picache, J. A.; Morris, C. B.; Codreanu, S. G.; May, J. C.; Sherrod, S. D.; McLean, J. A., Untargeted molecular discovery in primary metabolism: collision cross section as a molecular descriptor in ion mobility-mass spectrometry. Analytical chemistry 20**18,** *90* (24), 14484-14492.

Table S8. Level 1 metabolites detected in leaf (LF), wood (WD), bark (BK), root (RT), or heartwood (HW) samples of *V. harmandiana* using UPLC-IM-QTOFMS in ESI^+ and ESI^- modes.

Compound	Class	Adducts	Retention time (min)	Measured *m/z*	Mass Error (ppm)	$^{TW}CCS_{N2}$ (Å^2) in samples	ΔCCS%	Average Intensity (N = 3)					Previously identified?
								LF	WD	BK	RT	HW	
**Negative Mode**													
Protocatechuic acid	Benzene and substituted derivatives	$[M-H]^-$	2.10	153.0193	0.03	123.39	0.04	373.83	0.00	103.83	0.00	0.00	N
NAF-304	Naphthofurans	$[M-H]^-$	5.64	303.0886	3.97	163.21	2.20	0.00	0.00	301.17	0.00	0.00	N
XAN-330	Benzopyrans	$[M-H]^-$	8.03	329.0661	-1.68	172.68	0.27	0.00	257.98	77.75	530.33	1650.63	N
4-Hydroxyvertixanthone	Benzopyrans	$[M-H]^-$	7.83	299.0561	-0.16	164.44	0.11	34.00	161.75	128.08	167.49	407.03	N
1,3,6,7-Tetrahydroxyxanthone	Benzopyrans	$[M-H]^-$	3.90	259.0249	0.30	150.11	1.59	0.00	0.00	0.00	0.00	283.01	N
Quercetin-3-O-rutinoside (Rutin)	Flavonoids	$[M-H]^-$	2.56	609.1494	5.34	231.78	0.03	196024.49	527.08	1222.10	733.85	150.86	N
Kaempferol-3-O-rutinoside (Nicotiflorin)	Flavonoids	$[M-H]^-$	2.81	593.1543	5.28	230.22	1.36	57439.31	153.04	500.31	424.22	0.00	N
Isoquercitrin	Flavonoids	$[M-H]^-$	2.72	463.0899	3.56	201.01	1.02	9964.01	0.00	0.00	0.00	162.83	N
Chrysosplenetin	Flavonoids	$[M-H]^-$	6.56	373.0926	-0.84	193.62	2.27	0.00	0.00	428.04	0.00	0.00	Y
Eupatorin	Flavonoids	$[M-H]^-$	5.98	343.0828	1.37	177.78	2.34	0.00	0.00	0.00	0.00	137.91	N
(+)-Gallocatechin/(-)-Gallocatechin	Flavonoids	$[M-H]^-$	2.08	305.0666	-0.41	160.87	0.16	260.19	623.54	3689.71	2399.45	0.00	N
Quercetin	Flavonoids	$[M-H]^-$	4.16	301.0361	2.43	162.11	0.52	15443.28	413.70	352.50	0.00	0.00	N
Kaempferol	Flavonoids	$[M-H]^-$	4.97	285.0414	3.16	159.02	0.06	2542.71	4209.76	1658.43	3078.87	12467.76	N
Luteolin	Flavonoids	$[M-H]^-$	4.08	285.0407	0.68	159.02	0.30	0.00	0.00	81.05	0.00	0.00	N
Alpinetin	Flavonoids	$[M-H]^-$	4.83	269.0817	-0.71	164.08	0.47	120.36	0.00	0.00	0.00	0.00	N
Pinocembrin	Flavonoids	$[M-H]^-$	6.87	255.0663	0.02	157.49	0.77	129.55	117.78	0.00	0.00	304.17	N
Emodin-8-beta-D-glucoside	Anthracenes	$[M-H]^-$	4.02	431.0976	-1.80	197.59	0.97	0.00	1557.82	4281.12	1718.45	751.43	N
2,6-Dihydroxy-1,7,8-trimethoxy-3-methylanthraquinone	Anthracenes	$[M-H]^-$	5.07	343.0838	4.21	175.64	2.18	0.00	1722.95	1376.60	1998.66	9386.84	Y
Aurantio-obtusin	Anthracenes	$[M-H]^-$	5.98	329.0682	4.66	169.39	1.27	105.62	10237.59	13273.08	9340.74	22198.48	Y

Compound	Class	Adducts	Retention time (min)	Measured *m/z*	Mass Error (ppm)	$^{TW}CCS_{N2}$ (Å^2) in samples	ΔCCS%	Average Intensity (N = 3)					Previously identified?
								LF	WD	BK	RT	HW	
1-Hydroxy-6,7,8-trimethoxy-3-methylanthraquinone	Anthracenes	$[M-H]^-$	10.86	327.0871	-0.86	171.63	3.09	0.00	179.40	541.91	309.12	666.72	N
ATQ-314	Anthracenes	$[M-H]^-$	8.33	313.0729	3.66	166.39	0.50	64.70	3311.72	3550.24	4137.45	13293.52	N
2-Hydroxyemodin 1-methyl ether	Anthracenes	$[M-H]^-$	5.95	299.0573	4.10	163.30	0.89	0.00	4701.81	6678.55	2532.76	8787.33	Y
Obtusifolin	Anthracenes	$[M-H]^-$	8.03	283.0613	0.47	157.90	0.56	0.00	2008.25	2724.58	1623.29	8052.44	Y
Questin	Anthracenes	$[M-H]^-$	6.55	283.0611	-0.23	160.24	2.03	0.00	1139.94	2587.42	1997.44	3358.10	N
Emodin	Anthracenes	$[M-H]^-$	9.59	269.0468	4.76	152.28	0.19	107.61	9613.92	11649.87	6120.83	10509.31	Y
PNQ-334	Isochromanequinones	$[M-H]^-$	6.36	333.1343	-0.31	177.98	1.65	0.00	386.48	488.35	1877.77	10870.61	Y
PNQ-332	Isochromanequinones	$[M-H]^-$	7.81	331.1212	7.51	178.02	0.84	262.12	28466.91	33446.94	31219.06	69904.16	Y
PNQ-320	Isochromanequinones	$[M-H]^-$	8.19	319.1183	-1.42	171.80	1.84	0.00	1184.86	3237.98	1125.69	647.60	N
PNQ-318B	Isochromanequinones	$[M-H]^-$	10.28	317.1043	4.04	171.84	0.28	0.00	4129.52	9996.75	3557.74	1389.79	Y
PNQ-318A	Isochromanequinones	$[M-H]^-$	5.87	317.1055	7.74	171.84	1.43	105.47	16737.92	21993.97	15258.11	25593.88	Y
PNQ-302	Isochromanequinones	$[M-H]^-$	5.67	301.1111	9.81	170.01	0.06	0.00	24306.72	38742.18	33696.74	38642.77	Y
PNQ-290	Isochromanequinones	$[M-H]^-$	7.38	289.1084	0.73	163.55	0.35	0.00	989.42	1324.77	1292.63	355.01	N
PNQ-288B	Isochromanequinones	$[M-H]^-$	9.25	287.0950	8.53	162.45	0.60	0.00	26456.02	45379.66	29274.12	16135.05	Y
PNQ-288A	Isochromanequinones	$[M-H]^-$	4.77	287.0941	5.60	165.87	1.24	0.00	1841.08	3826.46	2674.19	10053.79	Y
**Positive Mode**													
XAN-330	Benzopyrans	$[M+H]^+$	8.04	331.0817	1.57	168.31	0.84	0.00	0.00	0.00	0.00	174.81	N
Quercetin-3-O-rutinoside (Rutin)	Flavonoids	$[M+H]^+$	2.56	611.1624	2.91	233.92	0.32	85856.45	0.00	171.23	0.00	0.00	N
Kaempferol-3-O-rutinoside (Nicotiflorin)	Flavonoids	$[M+H]^+$	2.81	595.1666	1.37	229.63	1.28	18151.45	0.00	0.00	0.00	0.00	N
Isoquercitrin	Flavonoids	$[M+H]^+$	2.72	465.1036	1.75	201.77	0.07	2413.66	0.00	0.00	0.00	0.00	N
Chrysosplenetin	Flavonoids	$[M+H]^+$	6.57	375.1079	1.16	186.58	0.81	0.00	0.00	490.56	0.00	0.00	Y
5,3'-Dihydroxy- 3,7,4'-trimethoxyflavone	Flavonoids	$[M+H]^+$	7.75	345.0968	-0.34	172.25	2.40	0.00	0.00	0.00	0.00	82.30	N
(+)-Gallocatechin/(-)-Gallocatechin	Flavonoids	$[M+H]^+$	2.07	307.0814	0.47	170.24	0.51	0.00	0.00	876.10	525.56	0.00	N
Quercetin	Flavonoids	$[M+H]^+$	4.16	303.0514	4.97	164.60	0.54	19313.83	176.43	0.00	0.00	0.00	N
5-Hydroxy-7,4'-dimethoxyflavone	Flavonoids	$[M+H]^+$	10.40	299.0919	1.63	164.69	2.38	0.00	0.00	407.58	0.00	0.00	N

Compound	Class	Adducts	Retention time (min)	Measured *m/z*	Mass Error (ppm)	$^{TW}CCS_{N2}$ (Å^2) in samples	ΔCCS%	Average Intensity (N = 3)					Previously identified?
								LF	WD	BK	RT	HW	
(Apigenin 7,4-dimethylether)													
Eriodictyol	Flavonoids	$[M+H]^+$	3.96	289.0709	0.93	164.94	0.35	0.00	78.12	142.84	0.00	0.00	N
Kaempferol	Flavonoids	$[M+H]^+$	4.96	287.0557	2.29	159.11	1.88	1993.99	1216.64	74.06	788.85	6785.30	N
Naringenin	Flavonoids	$[M+H]^+$	4.66	273.0757	-0.14	159.47	1.87	0.00	807.76	1016.13	1117.78	3853.08	N
5-Hydroxy-7-methoxyflavanone (Pinostrobin)	Flavonoids	$[M+H]^+$	10.23	271.0973	3.07	159.52	0.63	290.41	0.00	0.00	0.00	0.00	N
Alpinetin	Flavonoids	$[M+H]^+$	4.82	271.0968	1.08	159.52	0.43	139.60	0.00	0.00	34.85	0.00	N
Apigenin	Flavonoids	$[M+H]^+$	4.78	271.0607	2.15	156.52	0.04	0.00	124.95	1267.89	108.09	936.23	N
Pinocembrin	Flavonoids	$[M+H]^+$	6.87	257.0812	1.37	156.91	0.30	107.45	0.00	0.00	0.00	39.20	N
Pinocembrin chalcone	Flavonoids	$[M+H]^+$	6.55	257.0810	0.61	153.86	3.23	0.00	0.00	162.00	121.67	168.84	N
2,6-dihydroxy-1,7,8-trimethoxy-3-methylanthraquinone	Anthracenes	$[M+H]^+$	5.06	345.0973	1.10	172.25	1.31	0.00	2629.11	1679.32	2325.94	13259.17	Y
Aurantio-obtusin	Anthracenes	$[M+H]^+$	5.98	331.0829	5.09	166.88	0.22	0.00	4032.85	5203.01	3492.03	10826.36	Y
1-Hydroxy-6,7,8-trimethoxy-3-methylanthraquinone	Anthracenes	$[M+H]^+$	10.74	329.1021	0.33	169.76	0.23	0.00	190.79	564.48	803.72	1005.14	N
ATQ-314	Anthracenes	$[M+H]^+$	8.32	315.0874	3.53	164.34	0.25	0.00	954.59	1209.04	1355.64	5149.23	N
2-Hydroxyemodin 1-methyl ether	Anthracenes	$[M+H]^+$	5.96	301.0713	1.95	160.26	1.14	0.00	798.32	1242.81	403.70	1689.29	Y
Questin	Anthracenes	$[M+H]^+$	6.56	285.0760	0.86	157.66	0.51	0.00	396.22	891.91	733.25	1591.30	
Obtusifolin	Anthracenes	$[M+H]^+$	8.03	285.0759	0.64	156.16	0.12	0.00	143.16	200.14	104.83	865.44	Y
Emodin	Anthracenes	$[M+H]^+$	9.60	271.0605	1.31	153.47	0.22	0.00	140.20	157.06	0.00	174.60	Y
PNQ-346	Isochromanequinones	$[M+H]^+$	10.71	347.1490	0.28	177.73	0.54	0.00	256.80	1439.98	327.49	363.05	N
PNQ-334	Isochromanequinones	$[M+H]^+$	6.36	335.1489	0.04	175.22	0.24	0.00	0.00	0.00	267.54	2252.24	N
PNQ-332	Isochromanequinones	$[M+H]^+$	7.80	333.1358	7.59	173.88	0.55	492.28	20014.87	23352.19	20612.41	46019.49	Y
PNQ-318A	Isochromanequinones	$[M+H]^+$	5.86	319.1182	1.86	169.97	0.56	0.00	4425.58	6611.03	3391.16	5551.60	Y
PNQ-318B	Isochromanequinones	$[M+H]^+$	10.28	319.1181	1.38	169.97	0.28	0.00	111.18	352.36	0.00	0.00	Y
PNQ-302	Isochromanequinones	$[M+H]^+$	5.67	303.1244	5.75	167.48	0.31	0.00	2652.89	6755.77	5173.57	9358.55	Y
PNQ-288A	Isochromanequinones	$[M+H]^+$	4.76	289.1073	0.99	163.48	0.24	0.00	431.24	890.07	628.31	3407.54	Y

Table S9. Tentative metabolites identified in heartwood (HW), bark (BK), root (RT), wood (WD), or leaf (LF) samples of *V. harmandiana*.

No.	Tentative Metabolite	*m/z*	Retention time (min)	Experimental $^{TW}CCS_{N2}$ (Å^2)	ΔCCS%	Mass error (ppm)	Detected in					Putatively identified compound class
							HW	BK	RT	WD	LF	
1a	Hexadecanamide and isomers	256.2639	12.93	178.68	4%	1.54	Y	Y	Y	Y	Y	Fatty Acyls
1b		256.2639	13.36	178.68	4%	1.72	Y	Y	Y	Y	Y	Fatty Acyls
2	Octadecan-1-amine	270.3163	11.45	186.40	4%	2.81					Y	Organonitrogen compounds
3a	Hexadecasphinganine and isomers	274.2746	4.81	184.93	4%	2.09	Y	Y	Y	Y	Y	Organonitrogen compounds
3b		274.2755	4.96	184.93	4%	5.43	Y	Y	Y	Y	Y	Organonitrogen compounds
4a	1-Deoxy-3-dehydrosphinganine and isomers	284.2948	13.92	185.97	2%	0.2	Y	Y	Y	Y		Organooxygen compounds
4b		284.2949	13.55	185.97	2%	0.34	Y	Y	Y	Y		Organooxygen compounds
4c		284.2950	14.21	185.97	2%	0.57	Y	Y	Y	Y		Organooxygen compounds
5	1-Deoxysphinganine	286.3108	11.92	185.91	1%	1.32	Y	Y	Y	Y	Y	Organonitrogen compounds
6	(1E)-1-(4-Hydroxy-3-methoxyphenyl)-7-phenyl-1-hepten-3-one	311.1647	13.72	182.57	3%	1.63	Y	Y		Y	Y	Diarylheptanoids
7	Phytosphingosine	318.3009	5.05	194.25	2%	1.85		Y				Organonitrogen compounds
8	No candidate	321.3155	12.39	187.66				Y				
9	Pipericine	336.3260	13.08	201.45	3%	0.18		Y	Y			Fatty Acyls
10	13-Docosenamide	338.3427	12.39	205.14	4%	2.77	Y	Y	Y	Y		Fatty Acyls
11c	No candidate	338.4002	12.42	206.38						Y		
11d	No candidate	338.5574	12.42	205.14						Y		
12	Docosanamide	340.3580	14.83	208.78	4%	1.66	Y	Y	Y	Y		Fatty Acyls
13	10,12-Tetracosanedione	367.3564	13.02	209.42	1%	1.74	Y					Organooxygen compounds
14a	Tridodecylamine	522.5967	14.38	263.38	3%	1.09	Y	Y	Y	Y		Organonitrogen compounds
14b	Tridodecylamine	522.5968	14.63	263.38	3%	1.09	Y	Y				Organonitrogen compounds
